# Structure and Gating Behavior of the Human Integral Membrane Protein VDAC1 in a Lipid Bilayer

**DOI:** 10.1021/jacs.1c09848

**Published:** 2022-02-14

**Authors:** Eszter E. Najbauer, Kumar Tekwani Movellan, Karin Giller, Roland Benz, Stefan Becker, Christian Griesinger, Loren B. Andreas

**Affiliations:** † Department of NMR-Based Structural Biology, Max Planck Institute for Multidisciplinary Sciences, Am Faßberg 11, 37077 Göttingen, Germany; ‡ Life Sciences and Chemistry, Jacobs University of Bremen, Campus Ring 1, 28759 Bremen, Germany

## Abstract

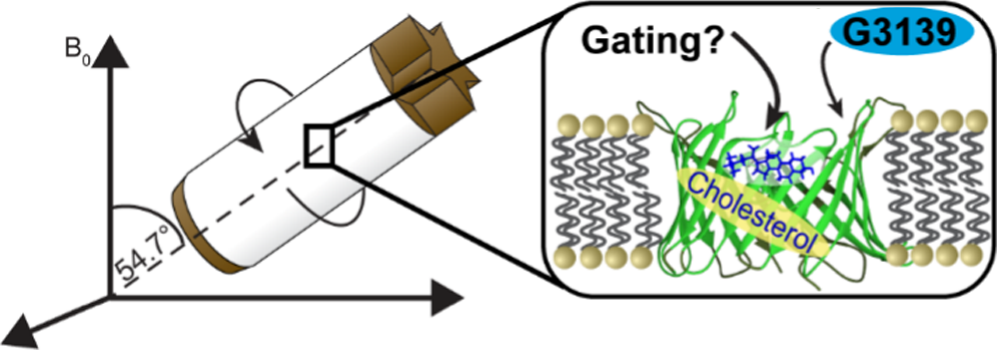

The voltage-dependent anion channel (VDAC), the most abundant protein in the outer mitochondrial membrane, is responsible for the transport of all ions and metabolites into and out of mitochondria. Larger than any of the β-barrel structures determined to date by magic-angle spinning (MAS) NMR, but smaller than the size limit of cryo-electron microscopy (cryo-EM), VDAC1’s 31 kDa size has long been a bottleneck in determining its structure in a near-native lipid bilayer environment. Using a single two-dimensional (2D) crystalline sample of human VDAC1 in lipids, we applied proton-detected fast magic-angle spinning NMR spectroscopy to determine the arrangement of β strands. Combining these data with long-range restraints from a spin-labeled sample, chemical shift-based secondary structure prediction, and previous MAS NMR and atomic force microscopy (AFM) data, we determined the channel’s structure at a 2.2 Å root-mean-square deviation (RMSD). The structure, a 19-stranded β-barrel, with an N-terminal α-helix in the pore is in agreement with previous data in detergent, which was questioned due to the potential for the detergent to perturb the protein’s functional structure. Using a quintuple mutant implementing the channel’s closed state, we found that dynamics are a key element in the protein’s gating behavior, as channel closure leads to the destabilization of not only the C-terminal barrel residues but also the α2 helix. We showed that cholesterol, previously shown to reduce the frequency of channel closure, stabilizes the barrel relative to the N-terminal helix. Furthermore, we observed channel closure through steric blockage by a drug shown to selectively bind to the channel, the Bcl2-antisense oligonucleotide G3139.

## Introduction

Mitochondrial function is essential for the viability of eukaryotic cells. Mitochondria are responsible for cellular respiration and also play a role in signaling pathways, cell growth and differentiation, and apoptosis.^1,2^ These processes all require a continuous flux of proteins,^3,4^ ions, and metabolites between the mitochondrial matrix and the cytosol through the mitochondrial membranes.

Voltage-dependent anion channels (VDACs)^5^ are integral membrane proteins abundant in the outer mitochondrial membranes,^6^ constituting up to 80% of the membrane surface.^7^ VDACs are the main avenues for the transport of various metabolites (e.g., adenosine diphosphate (ADP), adenosine triphosphate (ATP), pyruvate, malate) and ions (e.g., Ca^2+^)^8^ across the mitochondrial outer membrane,^9^ and have been suggested to form part of the mitochondrial permeability transition pore.^10,11^ (Note that despite “anion-selective” appearing in the name, VDAC also allows permeation of cations.)

VDAC exhibits a conductance and selectivity that is dependent on the membrane potential: the channel is in a high-conductance, moderately anion-selective state at low membrane potentials; however, at an absolute value of a few tens of millivolts, the channel closes and becomes cation-selective^5,12^ (cf. potential across the outer mitochondrial membrane is estimated to be in the range up to ±60 mV).^13^ VDAC is a critical player in mitochondrial apoptosis, and its malfunction may lead to cancer and neurodegeneration.^14^

**Figure 1:**
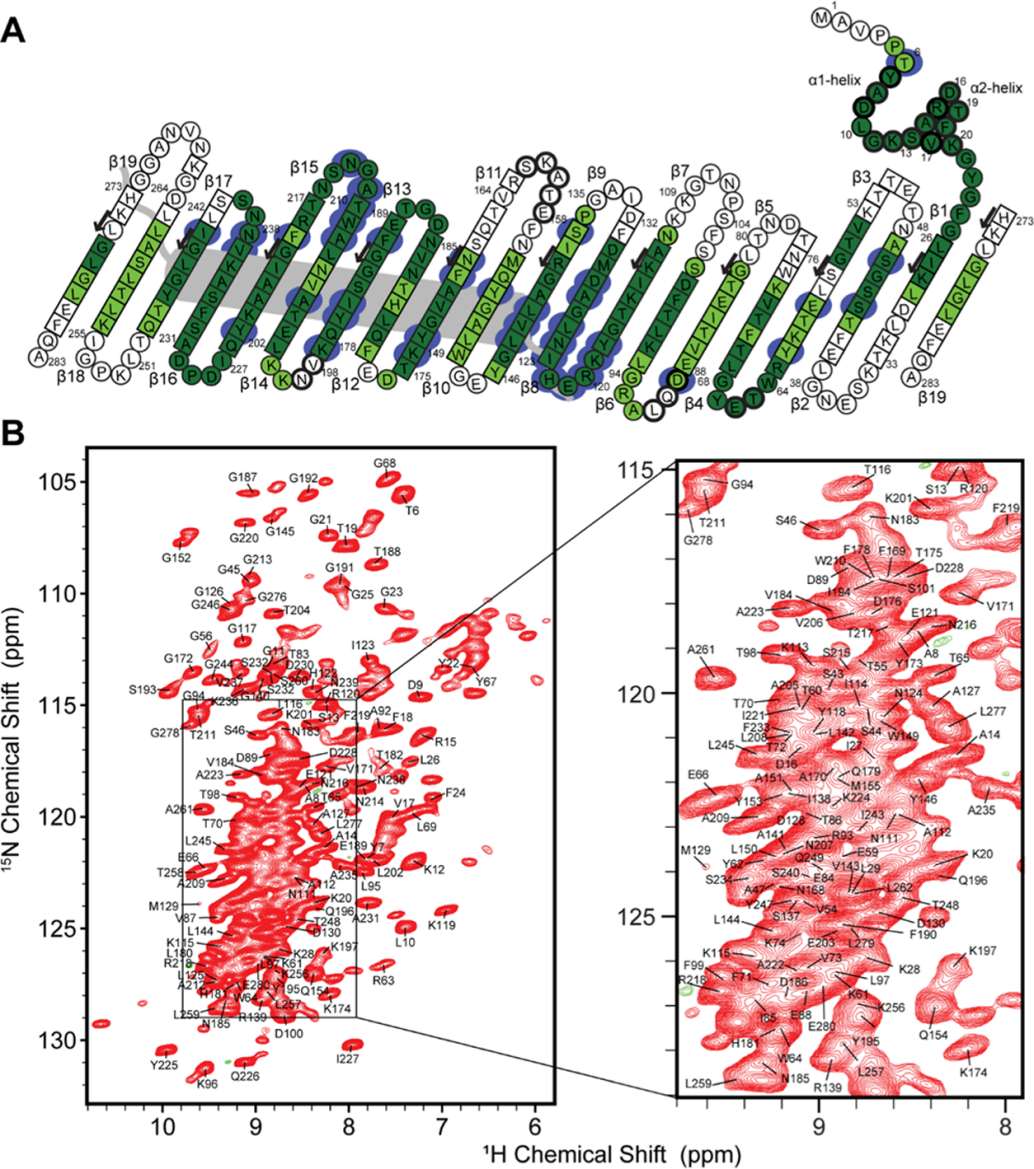
(A) Topology map of hVDAC1(E73V/C127A/C232S) in 1,2-dimyristoyl-*sn*-glycero-3-phosphocholine (DMPC) two-dimensional (2D) crystals showing assigned residues (dark green—automated assignments using FLYA,^76^ light green—manual assignments). Secondary structure predictions are based on TALOS-N (square—extended, circle—coil, thick circle—helical). When a residue was not assigned, sequence-based predictions are used. The topology depicted is based on the micelle structure of hVDAC1(E73V/C127A/C232S) (PDB: 5JDP). Residues K53, Q90, L91, K274, and L275 had confident automated assignments, but these were not confirmed manually and are not considered assigned. Blue coloring shows residues where chemical shifts measured in detergents^18,77^ and DMPC lipid bilayers show perturbations larger than 0.2 ppm (calculated from H, ^H^N, Cα, and Cβ shifts, weighted by 1, 0.17, 0.3, and 0.3, respectively). The position of the helix (as determined previously by solution NMR^18^) is shown in gray. (B) De novo assignments of hVDAC1(E73V/C127S/C232A) amides. The separation of peaks belonging to residues in the α-helix or the β-strands can be observed: most helical ^1^H shifts appear between 7 and 8 ppm, whereas residues in the β-strands tend to appear at higher ^1^H chemical shifts.

At 31 kDa in size, and comprising 283 residues, VDAC1 is the most abundant of the three known isoforms in humans.^15^ Human VDAC1 (hVDAC1) and mouse VDAC1 (mVDAC1) are closely related, differing by only four amino acids. Structures of hVDAC1 and mVDAC1 in detergent micelles^16−19^ and also from bicelles^20−22^ showed a β-barrel composed of 19 β-strands and an N-terminal α-helix positioned horizontally inside the pore, which in some structures^19,20^ is divided into two helical segments (α1 and α2) by residue G11 (Figures 1 and S1). These structures, however, are not consistent with the structures inferred previously through functional studies, which show the pore wall to consist of 16 β-strands^12^ or an α-helix and 13 β-strands.^23^

Several studies have shown that the presence of detergents may alter the structural and dynamic properties of proteins,^24−26^ which provides motivation for structural studies in a membrane environment.^27−30^ There is increasing evidence that VDAC only assumes its fully functional form in a lipid bilayer,^31^ however, obtaining structural information under such near-native conditions is challenging. Initial magic-angle spinning (MAS) NMR investigations using ^13^C detection on VDAC preparations in liposomes yielded assignments for the N-terminal helix and nine residues from three β-strands.^32^ These were later extended to include residues from seven β-strands^33^ using various isotopic labeling schemes and 2D crystalline preparations,^34,35^ where local order arises from the densely packed channels, similar to VDAC’s natural state.^7,33^ Previously reported MAS NMR spectra indicate that VDAC’s structure is stable across a range of phospholipid compositions, including 1,2-dimyristoyl-*sn*-glycero-3-phosphocholine (DMPC), 1,2-diphytanoyl-*sn*-glycero-3-phosphocholine (DPhPC),^34^ and a complex mixture of brain lipids.^36^

Membrane proteins of a size exceeding 30 kDa have long presented a challenge for MAS NMR spectroscopy, however, the optimization and refinement of proton-detected methods^37−43^ has made it possible to tackle systems of this size.^25,44,45^

The VDAC1 structures determined so far^16−20^ (Figure S1) are thought to represent the open state of the channel, which occurs in the absence of applied voltage. Models for describing the mechanism of channel closure include the unwinding^46^ or displacement^20^ of the whole α-helix to the center of the pore, a partial dislodging of the α2 helix from the barrel wall,^19,47^ and elliptic deformations (breathing motions) of the barrel.^46,48^ While a total dislocation of the helix from the barrel wall is improbable, as cross-linking experiments have shown that the L10C-A170C mutant (α1 helix-barrel cross-link) shows native-like gating behavior,^49^ a partial dislocation of the α2 helix may be possible, as the A14C-S193C mutant (α2 helix-barrel cross-link) shows an elevated preference for the open state.^50^ A direct determination of the gating mechanism has so far proved difficult due to technical challenges in applying voltage during structure determination with NMR spectroscopy or X-ray crystallography.

However, the closed state of the channel can also be implemented by mutations. The G21V/G23V mutant containing two mutations in the linker between the helix and the barrel has been shown by electrophysiology experiments to lock the channel into its closed state in around 50% of the channels.^51^ Apart from changes in membrane potential, VDAC’s gating behavior may also be regulated by the binding of various small molecules^19,52,53^ and proteins.^54,55^

We present the structure of hVDAC1 in a lipid bilayer. We obtained comprehensive assignments as well as the vast majority of structural restraints from measurements on a single sample from a set of three-dimensional (3D) and four-dimensional (4D) MAS NMR spectra using automated resonance assignment, and determined the protein’s structure from a combination of experimental structural restraints and modeling based on secondary structure prediction by TALOS-N.^56^

We also used a quintuple mutant of the protein building on the G21V/G23V mutant to implement the channel’s closed state to obtain insights into the channel’s gating mechanism. We additionally investigated the mechanism by which small-molecule interaction modulates gating behavior, as inferred from changes in NMR spectra, for the case of cholesterol and the Bcl2-antisense phosphorothioate oligonucleotide, G3139.

## Experimental Section

### Protein Expression

The expression of ^2^H, ^13^C, ^15^N-labeled and fully back-exchanged E73V/C127A/C232S hVDAC1 with a C-terminal (LE)-His6-tag (triple mutant) was based on the protocols of Malia and Wagner^57^ and Eddy et al.,^34^ with several modifications. Briefly, the protein was expressed in minimal medium (Table S1). Depending on the specific labeling scheme, the medium was supplemented with 1 g of ^15^N ammonium chloride and 4 g of ^13^C,^2^H d-glucose per liter as nitrogen and carbon source.

For producing perdeuterated hVDAC1(E73V/C127A/C232S), the bacteria were stepwise adapted to 100% D_2_O and the expression culture contained 100% D_2_O. A 1 L shaking culture was grown at 37 °C to an OD600 of 0.7–0.8 and expression was induced with 1 mM isopropyl β-d-1-thiogalactopyranoside (IPTG). The cells were harvested 20 h after induction. After cell lysis, inclusion bodies were isolated, dissolved in denaturing buffer (8 M urea, 50 mM Tris–HCl, pH 7.5, 100 mM NaCl, 20 mM imidazole, 0.5 mM phenylmethylsulfonyl fluoride (PMSF)), loaded on a 10 mL Ni^2+^ agarose column (Macherey-Nagel) and purified by elution with the same buffer supplemented with 250 mM imidazole. Fractions containing hVDAC1 protein were combined and dialyzed overnight against 4 L of precipitation buffer (50 mM Tris–HCl, pH 7.5, 50 mM NaCl, 1 mM ethylenediaminetetraacetic acid (EDTA)). The precipitated protein was dissolved in 25 mM sodium phosphate pH 7.0, 6 M guanidinium hydrochloride, 100 mM NaCl, 1 mM EDTA, and further purified by denaturing gel filtration on an SD75 16/60 gel filtration column (GE Healthcare). Subsequently the protein concentration was adjusted to 5 mg/mL and then refolded by dropwise dilution into a 10-fold volume of 4 °C refolding buffer (25 mM NaPi, pH 7.0, 100 mM NaCl, 1 mM EDTA, 1% lauryldimethylamine oxide (LDAO) (Anatrace)).

The refolded protein was dialyzed against a 20-fold volume of 4 °C cold 25 mM sodium phosphate, pH 7.0, 1 mM EDTA, 0.1% lauryldimethylamine oxide (LDAO), loaded on a 5 mL SP XL-cation exchange column (GE Healthcare) and eluted with a 60 mL linear gradient to 1 M NaCl in the same buffer. Subsequently, folded hVDAC1 protein was separated from misfolded or aggregated protein by a further gel filtration on an SD200 16/60 column (GE Healthcare) equilibrated with 25 mM sodium phosphate pH 7.0, 1 mM EDTA, 150 mM NaCl, 0.1% LDAO. The α proton exchange by transamination (α-PET) labeled sample was prepared as described previously.^58^

For spin labeling, an A2C mutation was introduced at the N-terminus of hVDAC1(E73V/C127A/C232S) and hVDAC1(G21V/G23V/E73V/C127A/C232S). During the purification steps, buffers contained 5 mM dithiothreitol (DTT); however, the pellet of the precipitation step (see the above paragraph) was washed with cold dialysis buffer containing no reducing agent. All subsequent purification steps were performed in the absence of reducing agents. After dissolving the pellet in the denaturing gel filtration buffer, *S*-(1-oxyl-2,2,5,5-tetramethyl-2,5-dihydro-1*H*-pyrrol-3-yl)methyl methanesulfonothioate (MTSL) was added in 10-fold molar excess and the solution was incubated for 2 h before the following gel filtration step. MTSL was once more added in 3-fold excess before refolding of hVDAC1. MTSL labeling was performed before refolding the protein to avoid any potential inaccessibility to tagging in the folded protein. As a control for potential misfolding, the MTSL tag was reduced with a 5-fold molar excess of ascorbic acid solution. To achieve this, a rotor fully packed with 2D crystalline hVDAC1(MTSL-A2C/E73V/C127A/C232S) was emptied into 200 μL of ascorbic acid solution (10 mM 2-(*N*-morpholine)ethanesulfonic acid (MES), pH 6.5, 150 mM NaCl, 20 mM MgCl_2_, 3 mM ascorbic acid) and incubated for 24 h at room temperature. The pellet was then washed three times each with 200 μL of the NMR buffer (10 mM MES, pH 6.5, 150 mM NaCl, 20 mM MgCl_2_), allowing diffusion of the buffer for a few hours between each wash, then packed into a rotor, and the spectrum was compared to hVDAC1(E73V/C127A/C232S).

### Two-Dimensional Crystallization of hVDAC1

The fractions containing the folded protein were combined and concentrated to about 3 mg/mL. 2D crystals were then prepared according to Eddy et al.^34,59^ Briefly, the purified protein was dialyzed overnight at 4 °C against 4 L of 50 mM Tris, pH 8.0, 0.6% (w/v) *n*-octyl polyoxyethylene (*n*-octyl-POE) (Bachem). Lyophilized DMPC (Avanti Polar Lipids) was dissolved in cold 50 mM Tris pH 8.9, 1% *n*-octyl-POE to a final concentration of 0.5 mg/mL. After adjusting the concentration of the dialyzed hVDAC1 protein to 1 mg/mL, equal volumes of the protein and the lipid solution were mixed and dialyzed at room temperature against 4 L of 10 mM MES, pH 6.5, 150 mM NaCl, 20 mM MgCl_2_, with four exchanges of the dialysis buffer over the course of 2 days, resulting in a lipid-to-protein ratio of about 25:1 by mole. After the last buffer change, the sample was dialyzed for 6 more days at room temperature. During this time, 2D crystals appeared. The crystals were finally collected by ultracentrifugation and transferred to a magic-angle spinning (MAS) NMR rotor.

The same protocol was used for preparing the G21V/G23V/E73V/C127A/C232A quintuple mutant implementing the channel’s closed state.

### Negative-Stain Electron Microscopy

Two-dimensional crystals of hVDAC1(E73V/C127A/C232S) were applied to 400-mesh copper grids (Plano, Wetzlar, Germany) and stained with 1% uranyl acetate (Merck). Images were acquired using a Philips CM120 electron microscope (Philips, Amsterdam, The Netherlands) equipped with a TemCam 224A slow-scan CCD camera (TVIPS, Gauting, Germany) at a defocus of 2.3 μm.

### NMR Spectroscopy

The ^31^P powder pattern (static) spectra were acquired on a 599 MHz Bruker Avance III spectrometer in a 1.3 mm rotor averaging 24576 scans for each spectrum. To eliminate baseline distortions, we implemented a Hahn echo before detection. The temperatures for the measurement (below and above the phase-transition temperature, 283 and 303 K, respectively) were calibrated using 100% methanol.^60^

All MAS spectra were recorded using proton detection and fast magic-angle spinning^61,62^ on a narrow-bore Bruker Avance III HD 800 MHz spectrometer in a three-channel probe (^1^H, ^13^C, ^15^N) at 55 kHz spinning frequency in a 1.3 mm rotor, with the VT gas flow set to 900 L/h at 250 K. The only exceptions were the (H)(CA)CB(CA)NH and (H)(CA)CB(CA)(CO)NH spectra, which were measured on a narrow-bore Bruker Avance III HD 950 MHz spectrometer in a four-channel 0.7 mm probe at 90.909 kHz MAS. For these two spectra, the VT gas flow was set to 300 L/h at 270 K. Typical 90° pulse lengths of 2.5 μs (^1^H), 3.1 μs (^15^N), and 4 μs (^13^C) were used for all measurements. Heteronuclear magnetization transfers were implemented using cross-polarization (CP). During evolution periods, 12.5 kHz two-pulse phase-modulated (TPPM) decoupling^63^ was used on ^1^H, and 10 kHz waltz-16 decoupling^64^ on both heteronuclei. Water suppression was achieved with the MISSISSIPPI^65^ scheme applied at 13.75 kHz. Recycle delays were set to 0.8–1.0 s.

The following 3D spectra were acquired: (H)CANH,^39^ (H)CA(CO)NH,^39^ (H)(CA)CB(CA)NH,^39^ (H)(CA)CB(CA)(CO)NH,^39^ (H)CONH,^39^ (H)(CA)CO(CA)NH,^39^ (H)N(CO)(CA)NH,^38^ (H)N(CA)(CO)NH,^38,66^ and H(H)NH.^67^ In addition, we acquired 4D spectra for assignment:^68^ (H)CACONH, (H)(CO)CACONH,^41^ structure determination:^69^ HN(H)(H)NH (2.3 ms RFDR^70^ mixing),^71^ and for mapping protein–water and protein–lipid contacts: H(H)(N)CANH (50 ms nuclear Overhauser effect (NOE) mixing).^67^ All spectra were referenced to the water signal at 4.7 ppm (using the IUPAC frequency ratios for DSS and liquid ammonia), assuming a sample temperature of 30 °C based on external calibration. For the multidimensional experiments (3D and above), typical acquisition times in the indirect dimensions were 20 ms on ^15^N, 10 ms on ^13^C, and 5.3 ms in the indirect ^1^H dimension. Experimental parameters are summarized in Table S2. Additionally, (H)NCAH^72,73^ and (H)NcoCAH^72,73^ spectra were acquired for the α-PET labeled sample.

D_2_O exchange was investigated by washing the sample twice with 200 μL of deuterated buffer, then packing the rotor and measuring (H)CANH experiments immediately afterward. The (H)CANH spectrum was recorded in a time frame of 1 day.

Spectra were corrected for magnetic field drift using a Topspin AU macro,^74^ and for longer acquisitions, blocks of ∼1-day acquisition were added. Spectra were processed using 10 ms of data in the direct ^1^H dimension, and in indirect dimensions, all acquired data were used (as specified in Table S2, usually 20 ms on ^15^N and 10 ms on ^13^C). The data were processed in Topspin 3.5pl7 using a squared sine window function with a phase shift of π/2 and analyzed in Sparky 3.113.^75^

### Ligand Binding

To test cholesterol binding, a DMPC/cholesterol/protein ratio of 25:5:1 was used and the same 2D crystalline sample preparation protocol was applied, The lipid was deuterated on the acyl chains (*d*_54_-DMPC). The H(H)CANH spectrum was recorded with the CP-based pulse sequence described in Najbauer et al.,^67^ replacing the H–N and N–C CP steps with one H-CA CP step. The CP conditions are identical to those described in Table S2 for the (H)CANH experiment. The NOE mixing time was 75 ms. The spectrum was recorded using 3.55% nonuniform sampling with 5.2 ms indirect ^1^H, 10.1 ms ^13^C, 20.1 ms ^15^N, and 21.3 ms direct ^1^H evolution times, averaging a total of 38 scans.

G3139 (Sigma-Aldrich) was added to the reconstituted membrane protein sample resuspended in 200 μL of sample buffer at an estimated 1:1.5 protein/ligand molar ratio. (H)NH, (H)CANH, and (H)(CO)CA(CO)NH spectra were recorded with the parameters described previously.

### Resonance Assignment

Automated assignments were obtained from the FLYA module of the CYANA software package^76^ using a custom library file that included proton-detected MAS experiments (Supporting Information, SI). Tolerances were set to 0.07 ppm for ^1^H and 0.4 ppm for heteronuclei. For each of the 10 runs, the initial population was set to 50, and 15 000 iterations were performed. A resonance was counted as confident, when it converged to the same assignment in at least 80% of the runs. Automated assignments were then checked and extended manually. Chemical shifts can be accessed under BMRB ID 34694.

The closed-state G21V/G23V/E73V/C127A/C232S quintuple mutant and the ligand-bound samples were assigned based on (H)CANH and (H)(CO)CA(CO)NH spectral pairs and resonances of the E73V/C127A/C232S mutant.

Chemical shift perturbations (CSP) between the triple mutant in LDAO detergent^77^ and a DMPC lipid bilayer (this work) were calculated from the nuclei indicated as a combination of chemical shift changes (Δδ_*i*_) with weighting factors (α*_i_*) of 1 on ^1^H, 0.17 on ^15^N, and 0.3 on ^13^C.^78^
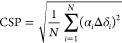


### Structure Calculation

Structure calculation was done with CYANA 3.98.13.^79^ Unambiguous distance restraints were assigned in the HN(H)(H)NH spectra, and hydrogen bonds were inferred from these for the structure calculation. Dihedral angle restraints were generated from chemical shifts using the TALOS-N web server.^56^ Error margins were set to 3 times the predicted standard deviations, capped at a minimum of ±30°. Due to clashes in the loop between β3 and β4, torsion angles of residues T65-G68 were removed. Due to clashes, error margins of the torsion angles of T211 and R218 were set to 4 times the predicted standard deviations. Additional hydrogen bonds were modeled in between two residues predicted to be in extended conformation based on either chemical shifts or the primary sequence. A residue was also considered to be in extended conformation if both its neighbors were predicted to be extended. The barrel’s diameter was defined based on atomic force microscopy (AFM) measurements in a lipid bilayer to be between 27 and 38 Å.^7^ The position of the helix was defined using both long-range-distance restraints from previous MAS NMR measurements in a lipid bilayer and MTSL labeling. Helix-barrel contacts used for structure calculation from previous studies were: L10 Cγ-V143 Cα, Cβ,^32^ and A14 Cβ-S193 Cβ.^80^ For MTSL distance restraints, an upper limit distance restraint of 10 Å was implemented for peaks where *I*_rel_ < 0.1 *I̅*_rel_, where *I*_rel_ = *I*_MTSL_/*I*_unlabeled_. (Distances were defined as the distance between A2Cβ and the corresponding amide proton.) These residues were R120-N124. An additional four residues toward the C-terminus of the barrel (G145, K174, T217, and N238) met the criterion for strong signal attenuation but were not used for distance restraints, as they did not cluster together, and their bleaching most probably stems from MTSL labels in neighboring barrels. We noticed slow convergence toward low target function values such that many structures of the standard CYANA bundle had high associated target function values above 10. Therefore, many structures were calculated, a total of 2400, and the 10 structures with the lowest target functions were selected. (Two structures not compatible with a planar lipid bilayer were excluded.) Distance restraints included in the structure calculation are summarized in Table S4. All restraint files can be found in the Protein Data Bank (PDB ID7QI2).

### Electrophysiology Measurements

Planar phospholipid bilayer measurements of the hVDAC1(G21V/G23V/E73V/C127A/C232S) mutant were performed as described previously.^12^ Lipid bilayers of the Mueller–Rudin type^81^ were created by painting 5 μL of a solution of 1% DPhPC lipids in *n*-decane over the hole (area ≈ 0.4 mm^2^) of a Teflon cuvette. A 1 M KCl solution containing 10 mM *N*-(2-hydroxyethyl)piperazine-*N*′-ethanesulfonic acid (HEPES), pH 6.0, was used as an electrolyte. The hVDAC1 mutant in LDAO was diluted with 1% (w/v) Genapol to a final concentration of approximately 5 μM and mixed 1:1 with cholesterol powder suspended in 1% (w/v) Genapol. Aliquots of hVDAC1 (typically 1–5 μL) were added to both cuvette chambers at a standard membrane potential of 20 mV. Current was measured with Ag/AgCl electrodes and an in-house amplifier.

## Results

### Sequential Assignment

Using the protocol developed by Dolder et al.^35^ and adapted for MAS NMR measurements by Eddy et al.,^34^ a 2D crystalline sample of E73V/C127S/C232A-hVDAC1 was prepared. The three mutations were introduced to improve sample stability (preventing oligomerization through the removal of the two cysteines) and to improve spectral quality. These mutations, however, do not influence VDAC’s gating behavior: Aram et al. had previously shown that the two cysteines in VDAC are not required for voltage gating,^82^ nor is glutamine required at position 73.^83^ Using negative-stain electron microscopy, a lamellar structure was observed, showing that our preparation reproduced the microscopically ordered layers described by Eddy et al. in their preparation (Figure S2).^34^ The ^31^P powder patterns of the 2D DMPC lipid crystals also confirm that the lipids in the sample are lamellar (Figure S3).

To achieve an assignment as complete as possible of hVDAC1 in lipids, a set of three- and four-dimensional assignment experiments were recorded on a perdeuterated, 100% H^N^ back-exchanged 2D crystalline sample of hVDAC1(E73V/C127S/C232A), following the proton-detected strategy described in several previous publications (Figure S4).^39,42^ A full list of spectra and the experimental parameters are shown in Table S2.

Peak lists from the spectra listed in Table S3 were used as an input for the FLYA algorithm of the CYANA software package, which uses a genetic algorithm to assign proteins in an automated manner, avoiding the bias of human spectral evaluation.^76^ To test for the usefulness of the information provided by different spectra in the assignment of membrane proteins, different subsets of assignment spectra were used as an input to FLYA. Peak lists from only the six 3D experiments linking Cα, C′, and Cβ (“basic six”) yielded confident assignments for 74 residues, the algorithm performing particularly well for the majority of the α-helix (residues 5–21), as well as residues from β-strands either containing many residues with distinct chemical shift values (β2) or strands with particularly large chemical shift dispersion of the peaks (e.g., β15, β16) (Figure 1A, dark green). (Assignments where the residue was part of an assigned stretch of at least two residues with at least two assigned backbone atoms were considered confident.)

We found that the most assignments were gained by including information from either 4D (H)COCANH and (H)(CO)CACONH spectra or the 3D (H)N(CO)(CA)NH and (H)N(CA)(CO)NH spectra. Including information from either of these two spectral pairs besides the “basic six” experiments roughly doubled the number of confident assignments: 150 and 137 residues were assigned, respectively. Little additional information was to be gained using peak lists from additional spectra, achieving 163 confident assignments in total (Figure 1A, dark green), when including peak lists from 13 different spectra. The assignment levels achieved using different subsets of spectra are shown in Table S3. Assignments were manually checked and extended to a total of 194 out of 283 residues, as shown in Figure 1A (light green) and Figure 1B. Two additional AG spin systems were identified, assignable presumably to A134-G135 and A270-G271; however, reliably linking these to other spin systems was not possible. Residues in the α-helix were particularly easy to assign, having high signal intensities and good separation from the predominantly β-sheet resonances of the protein. This is in agreement with previous observations by MAS NMR in lipid bilayer samples where assignment of the α-helix was possible,^32,33^ and in contrast to studies in detergents, where the protein’s N-terminus was completely^16^ or partially^19,48^ unassignable. Assuming the topology of the protein to be identical to that in detergents, our assignments provide a comprehensive overview of the protein with residues assigned from the N-terminal α-helix and all 19 β-strands. To pinpoint any differences between hVDAC1 structure in detergents and in lipids, we calculated chemical shift perturbations between assignments of hVDAC1(E73V/C127A/C232S) in 2D DMPC lipid crystals and in LDAO micelles. Chemical shifts matched well overall, with 41 residues showing perturbations larger than 0.2 ppm, the largest values being 1.15 ppm (F169), 0.70 ppm (Y62), and 0.65 ppm (R120 and A134). These 41 residues are located almost exclusively in strands, which have the helix running next to them, close to the helix-barrel contact sites determined by solution NMR (Figures 1A and S5). The loop between strands β7 and β8, where two hydrogen bonds between the carbonyl oxygens of A2 and P4 and the H^N^ of H122 and the Nδ2 of N124 were identified by X-ray crystallography,^20^ was particularly affected by the change of environment.

The chemical shift perturbations could be a result of helix-barrel contacts being influenced by the binding of detergent molecules, which are known to bind to hydrophobic regions.^84^ The particularly large H^N^ and N^H^ chemical shift changes of residues I123 and N124 (H^N^: 0.32 ppm, and 0.53 ppm, respectively, N^H^: 4.9 ppm in both cases) could be an indication of not only altered hydrogen bonding between the protein’s N-terminus to the loop^78^ but also a conformational change in this protein region; significant Cα and Cβ perturbations are observed for R120 Cβ: 3.2 ppm, I123 Cα: 1.3 ppm, and N124 Cβ: 1.5 ppm.

### Mobility in the Open Channel

**Figure 2:**
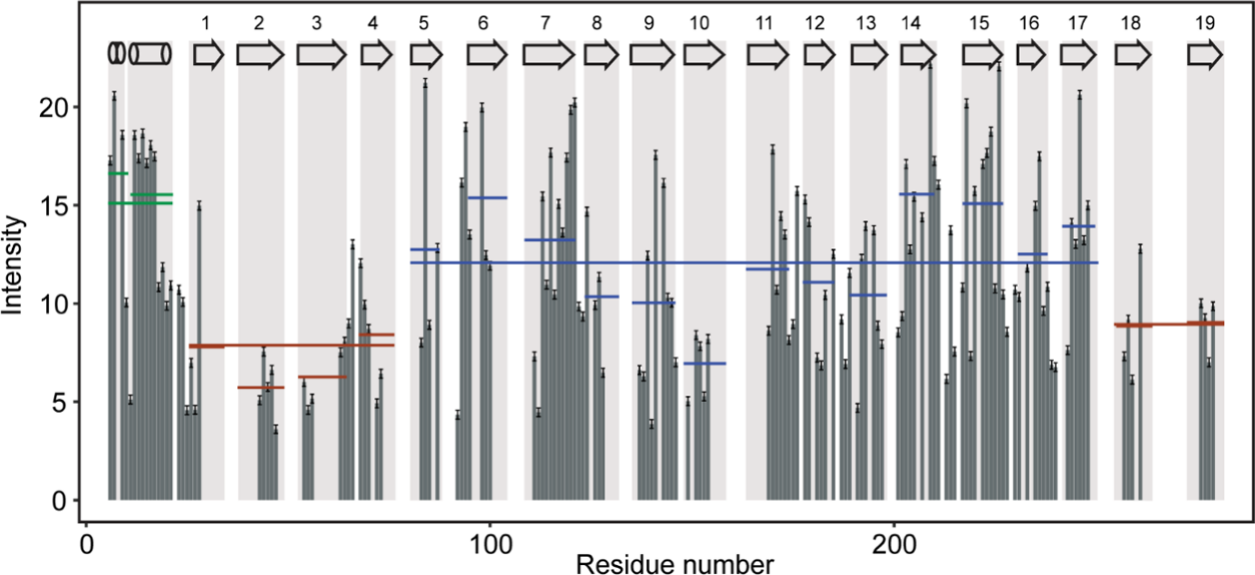
Signal intensities of hVDAC1(E73V/C127A/C232S) in a lipid bilayer as determined from the (H)CANH spectrum. Gray rectangles indicate secondary structure elements as determined previously in micelles.^18^ For each secondary structure element (α-helical segment or β-strand), the average peak intensities in that motif are shown as colored lines and the average peak intensities are also indicated for the whole region (including loops). Green shows average intensities in the helical region, red in the four N-terminal β-strands and the two C-terminal strands connecting to them, and blue shows average intensities in the rest of the β-barrel.

Peak intensities in MAS–NMR are excellent indicators of dynamics, as molecular motion can manifest as weaker dipolar coupling strength, resulting in a decrease of signal intensity.^85^ Motion on the μs timescale has been shown to interfere with magic-angle spinning,^86^ resulting in signal loss.^87^ We first characterized peak heights for the open-state hVDAC1(E73V/C127A/C232S) mutant. Similar to previous studies in detergents,^48^ we found a low signal intensity indicating increased mobility in the four N-terminal β-strands β1–4, as well as the two connecting C-terminal strands β18–19. With the exception of β10, strands β5–17 all showed higher average signal intensities, as did the α-helix (Figure 2). This is in agreement with H/D exchange experiments (Figure S6), where residues in strands β5–18 showed reduced rates of exchange. Helical residues showed the highest average intensity in the protein, indicating a well-structured α-helix,^32^ the α2 helix being somewhat more mobile than the α1 segment, as also observed in detergents.^19^

### Structure Calculation

We obtained unambiguous long-range amide–amide contacts between hydrogen-bonded residues from the four-dimensional HN(H)(H)NH spectrum (Figures S7 and S8), where mixing of magnetization between protons was achieved using radiofrequency-driven recoupling (RFDR).^70^ Direct helix-to-barrel contacts were not identified, due primarily to perdeuteration of the protein. To locate the helix, we introduced an N-terminal MTSL spin label at the A2C position (Figure S9), which resulted in five restraints to residues R120-N124 in the loop region between strands β7 and β8 (Figure S10). We also used helix-barrel contacts previously determined on fully protonated hVDAC1 in lipid bilayers, specifically L10 Cγ-V143 Cα, Cβ,^32^ and A14 Cβ-S193 Cβ.^80^

Contacts from the HN(H)(H)NH spectrum clearly defined the channel’s topology as a 19-stranded β-barrel and an N-terminal α-helix; however, to better define the barrel, especially around loop regions, we modeled additional hydrogen bonds based on secondary structure predictions from TALOS-N. Hydrogen bonds were modeled in between two residues predicted to be in extended conformation either based on chemical shifts or the primary sequence. Results of the TALOS-N prediction and hydrogen bonds identified in the HN(H)(H)NH spectrum and through modeling are shown in Figure S11.

Moreover, as the barrel is quite large and can exhibit elliptic deformations,^18^ we defined its shape based on AFM data recorded in lipid bilayers to have a diameter between 27 and 38 Å^7^ to improve convergence of structure calculation. To this end, we defined the distance of residues in the middle of the barrel, found in opposing strands (e.g., β1 vs β9 and β10) to be in the range of 27 and 38 Å. Residues in the middle of β-strands, a quarter of a barrel (five strands) away were defined to be 19.1–26.9 Å apart. All constraints used for structure calculation are listed in Table S4.

**Figure 3:**
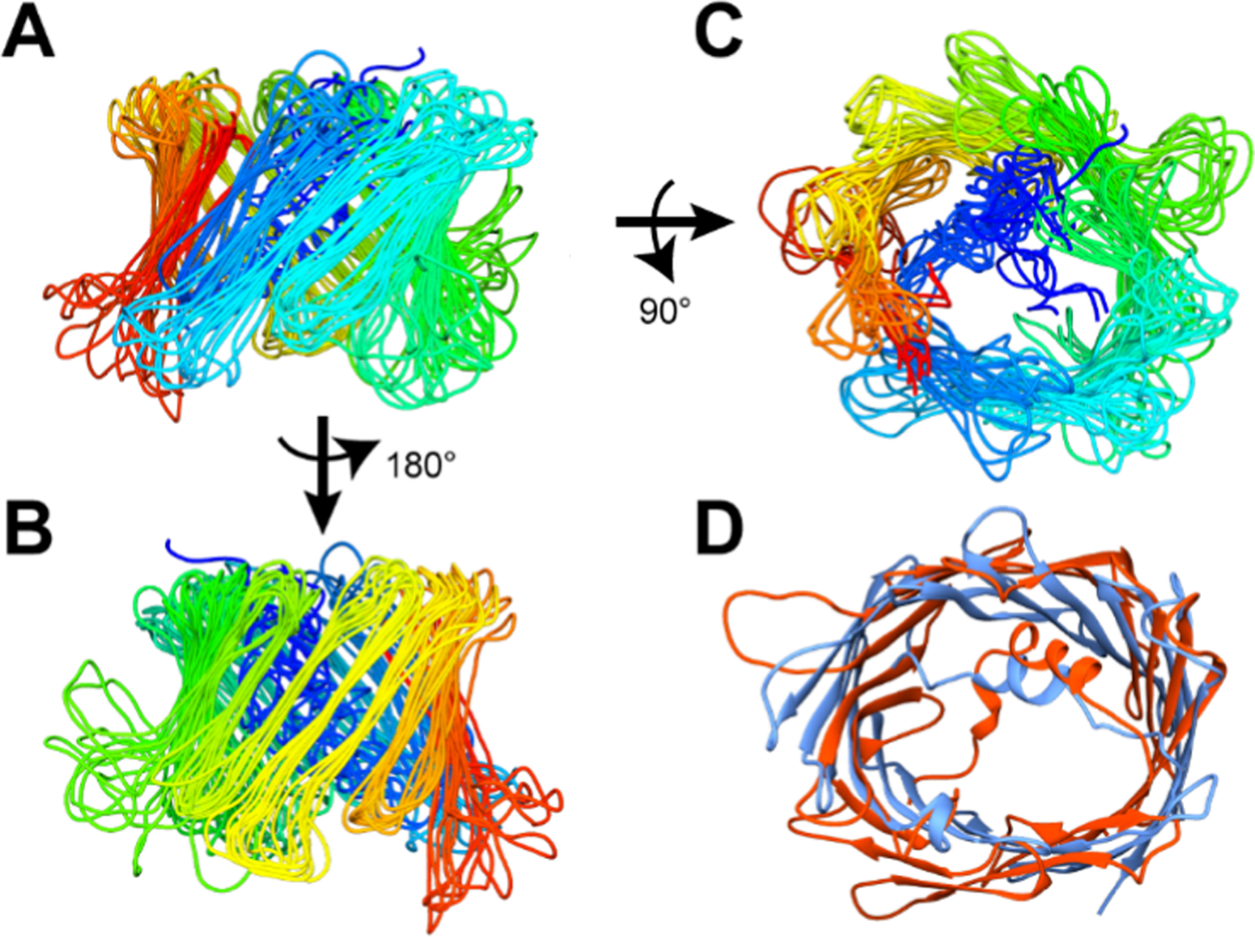
Structure of hVDAC1(E73V/C127A/C232S) in a DMPC lipid bilayer (A) viewed from the side, strands β1–10, (B) viewed from the opposite side, strands β10–19, and (C) viewed from above the barrel. Shown are the backbone traces of the 10 conformers with the lowest CYANA target functions excluding those incompatible with a lipid bilayer environment. The structures were aligned using the following residues in the α-helix and in β-strands: 6–9, 13–22, 27–32, 40–47, 55–63, 70–77, 83–87, 97–101, 112–118, 123–131, 137–144, 149–156, 168–174, 179–185, 189–199, 202–210, 218–225, 232–237, 243–250, 256–264, 272–280. (D) Superposition of the structure of hVDAC1(E73V/C127A/C232S) in DMPC lipid bilayer (red) and the high-resolution NMR structure in LDAO micelles (PDB: 5JDP, blue). The structure closest to average has been selected from both ensembles using the WHAT IF server.^88^

From the ensemble of lowest-energy structures, we excluded topologies where some strands form an angle relative to the rest of the barrel (Figure S12). These “flipped” strands contained residues previously shown to be in contact with lipids,^67^ which is inconsistent with this topology and the fact that hVDAC1 is an integral membrane protein. The root-mean-square deviation (RMSD) of the backbone excluding loops is 2.2 Å. The ensemble of the 10 lowest-energy structures excluding the above-mentioned topology is shown in Figure 3A–C, and the superposition of the structure closest to average in a DMPC lipid bilayer and in LDAO micelles is shown in Figure 3D. Since RMSD is not directly comparable to the resolution obtained from X-ray crystallography, we used the resolution-by-proxy (ResProx) method^89^ to predict the atomic resolution of our NMR structure. Using the same models with which RMSD was calculated, we obtained an estimated resolution of 2.7 ± 0.1 Å for our MAS NMR structure. Statistics of structure calculation are summarized in Table 1.

**Table 1:** Structure Calculation Statistics^a^

restraints	
total (HN(H)(H)NH)	97
short-range (|*i* – *j*| ≤ 1)	24
medium-range (1 < |*i* – *j*| ≤ 5)	13
long-range (|*i* – *j*| ≥ 5)	60
H-bond restraints (manual)	61
H-bonds (modeled)	15
MTSL restraints	5
previous MAS NMR data	3
dihedral angle restraints (ϕ and ψ)	292
AFM-based restraints	38
target function, average (Å^2^)	5.10
min/max	3.43/6.67
average RMSD to the mean (Å)	
backbone RMSD	2.2
heavy atom RMSD	2.8

a The RMSD was calculated using the following residues, belonging to structured regions in the protein (α-helix, β-strands): 6–9, 13–22, 27–32, 40–47, 55–63, 70–77, 83–87, 97–101, 112–118, 123–131, 137–144, 149–156, 168–174, 179–185, 189–199, 202–210, 218–225, 232–237, 243–250, 256–264, 272–280.

### Characterization of Mobility in Closed-State hVDAC1

**Figure 4:**
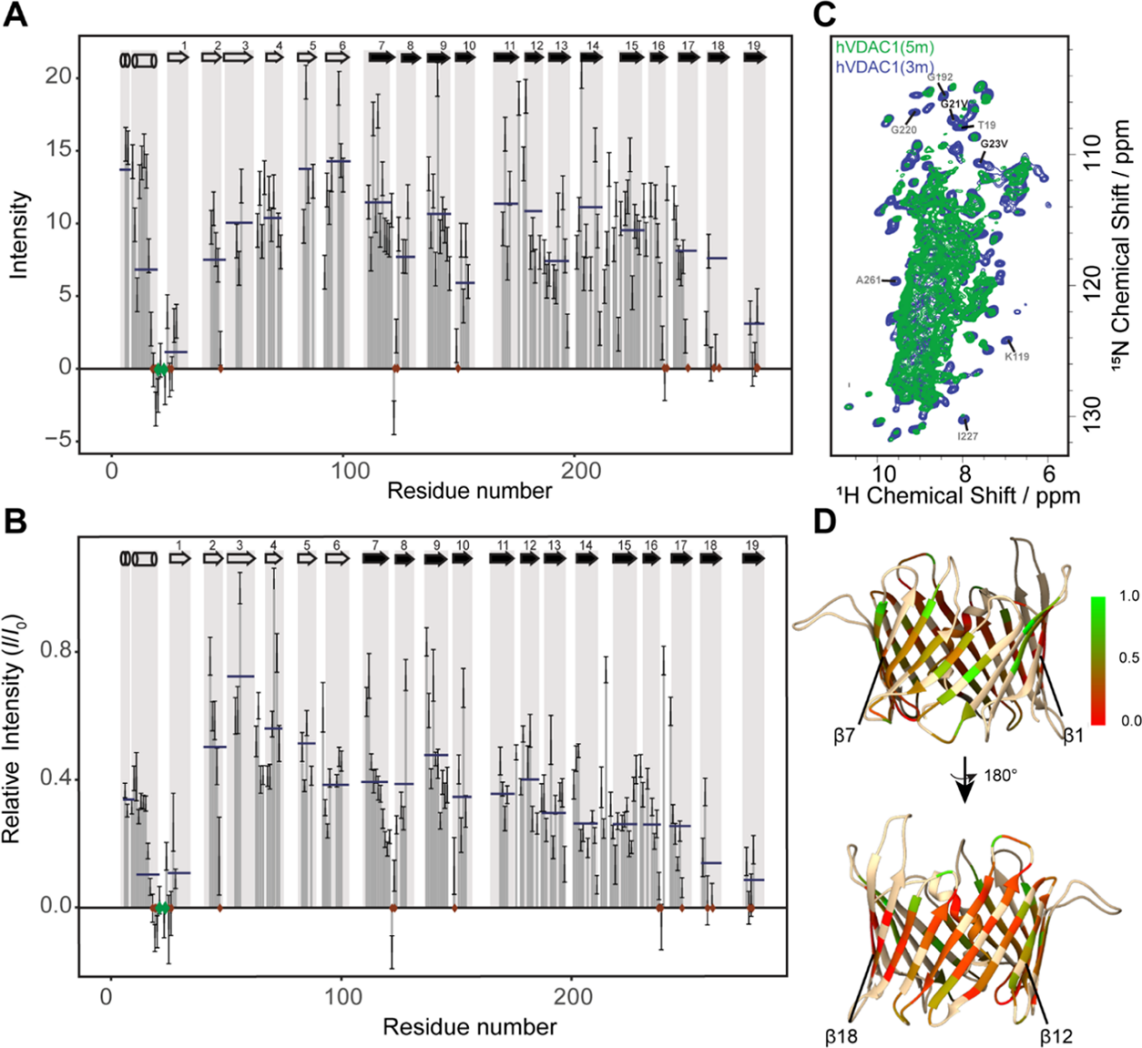
Closure of the hVDAC1 channel—effect of the G21V/G23V mutations. (A) Peak intensities in the (H)CANH spectrum of the hVDAC1(G21V/G23V/E73V/C127A/C232S) quintuple mutant. (B) Relative intensity changes in the (H)CANH spectra upon introduction of the G21V/G23V mutations (*I* denotes intensities of the quintuple hVDAC1(G21V/G23V/E73V/C127A/C232S) (5m), and *I*_0_ denotes the intensities of the hVDAC1(E73V/C127A/C232S) triple mutant (3m)). Relative intensities are scaled such that the greatest value is 1 (scaling factor: 0.41). Overlapping residues are not plotted in (A) or (B) due to the inaccuracy of intensities. Green diamonds show the position of the two mutations, and red diamonds mark residues where the peak’s intensity in the quintuple mutant dropped below 2σ, where σ is the rms noise level. Horizontal blue lines show the average relative intensities in the N- and C-terminal parts of the α-helical region (separated by the kink at G11), as well as in the β-strands. β-strands with the helix running in front are marked by black arrows. (C) Overlay of the (H)NH spectra of the closed-state quintuple (5m) (green) and the open-state triple mutant (3m) (blue). The peaks of G21 and G23 mutated to V are labeled in black, and as an example some residues disappearing from the spectrum have been assigned (gray). (D) Relative intensities plotted onto the lipid bilayer structure of hVDAC1(E73VC127AC232S). Red corresponds to a relative intensity of 0, and green corresponds to a relative intensity of 1. The side of the barrel in contact with the helix is shown on the bottom.

Investigation of hVDAC1’s gating mechanism and determining its closed state pose a problem for all structural biology methods, as the measurement would have to take place under applied voltage. This can be circumvented however with the investigation of hVDAC1 mutants permanently exhibiting the channel’s low-conductance state (Figure 4). Such a mutant was initially suggested by Geula et al.,^51^ where the G21V/G23V mutations were shown to partially lock the channel into a closed-state conformation. These mutations served as a basis for developing the closed-state G21V/G23V/E73V/C127A/C232S quintuple (5m) mutant by introducing mutations necessary for sample stability. The electrophysiology of this mutant was shown to be identical to that of the G21V/G23V mutant^77^ (Figure S13A,B).

The quintuple (5m) mutant’s (H)NH spectrum was found to overlay excellently with that of the triple (3m) mutant, with no significant changes in chemical shifts, and as expected, signals belonging to G21 and G23 could not be observed. It was however quite surprising that in the quintuple mutant’s (H)NH spectrum, many peaks were of low relative intensity or had disappeared entirely from the spectrum (Figure 4C). An (H)CANH spectrum was recorded to quantify any changes in intensity. Interestingly, peaks from the α2 helix and strands β7–17 were no longer higher in intensity than the rest of the barrel (Figure 4A). The region with the highest relative intensities included strands β2–5 (located on the opposite side of the barrel, where the N-terminal helix runs), while β18 and 19, as well as the C-terminal part of the helix (α2), and the neighboring β1 were almost completely broadened beyond detection. β7–17, the strands neighboring the helix in the open-state structure, were also significantly reduced in intensity, as well as the N-terminal part of the helix (α1) (Figure 4B,D).

### Investigation of Cholesterol Binding to hVDAC1

**Figure 5:**
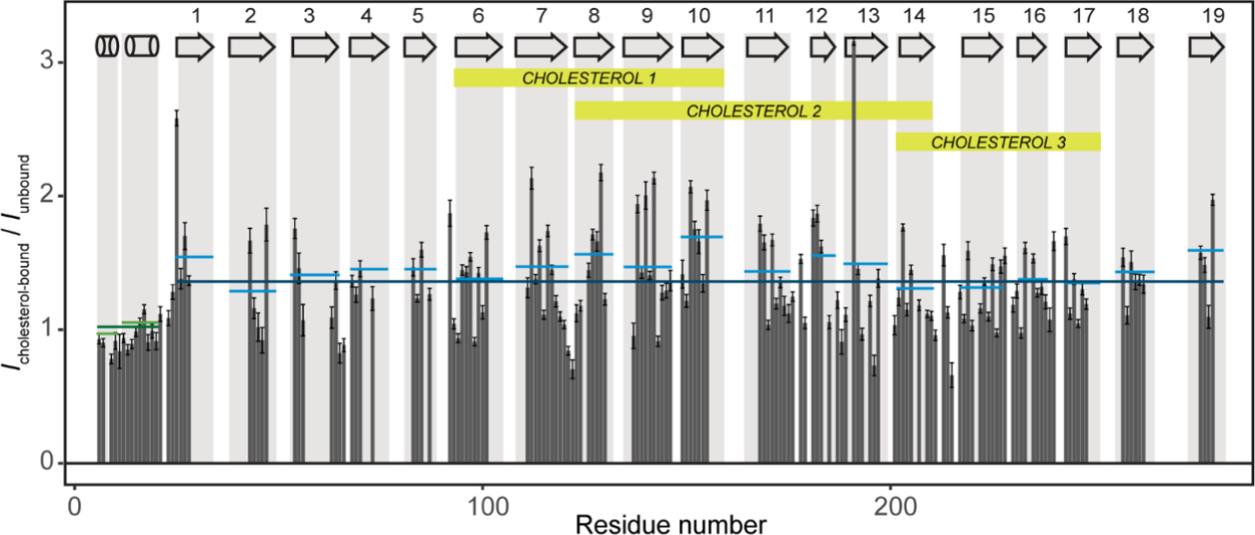
Change of peak intensities upon binding of cholesterol to hVDAC1(E73V/C127A/C232S) at a 1:5 protein/cholesterol molar ratio. Relative intensities (*I*_cholesterol-bound_/*I*_unbound_, no scaling applied) are shown as a function of residue number. The average relative peak intensity of each secondary structural element is shown in colored lines: green—α-helix, blue—β-strand. The average relative peak intensity for the whole of the N-terminal helical region, as well as the barrel is shown as longer, dark green, and dark blue horizontal lines, respectively. Secondary structure elements are indicated with cylinders (α-helix) and arrows (β-strand) in the gray rectangles showing the extent of each structural element. The extent of the cholesterol binding sites, as determined by *z*–*z* mixing experiments,^36^ is marked in yellow. The indicated three cholesterol binding sites correspond to those identified previously in DMPC bilayers by MAS NMR.^36^

Since cholesterol was in some cases necessary to obtain normal function of recombinant hVDAC1^16^ and has been shown to bind strongly to the channel,^90^ we previously investigated the interaction of cholesterol and hVDAC1 in a lipid bilayer and identified three of the binding sites predicted through docking.^36,91^ Electrophysiology experiments have shown the binding of cholesterol not only to facilitate hVDAC1’s membrane insertion but also to slightly reduce the % closure ((1 – *G*_50 mV_/*G*_0_)100) of the channel.^52^ To understand whether cholesterol stabilizes the channel’s open state by reducing barrel mobility that is crucial for channel closure, we plotted the relative intensities (*I*_cholesterol-bound_/*I*_unbound_) measured in the (H)CANH spectrum of the cholesterol-bound sample and in the unbound sample (Figure 5). We found that relative signal intensities of residues in the barrel averaged ∼1.5-fold higher than the N-terminal helix, a clear indication of cholesterol’s stabilizing effect on barrel dynamics. Furthermore, the relative intensities of β7–13, strands with six of the seven identified cholesterol contacts, were all somewhat higher than average (Figure 5). This is in agreement with our observation that most probably binding sites 1 and 2 are more populated than site 3,^36^ and the cholesterol bound to these two sites stabilizes the contacting β-strands.

### Determining the Binding Site of G3139

**Figure 6:**
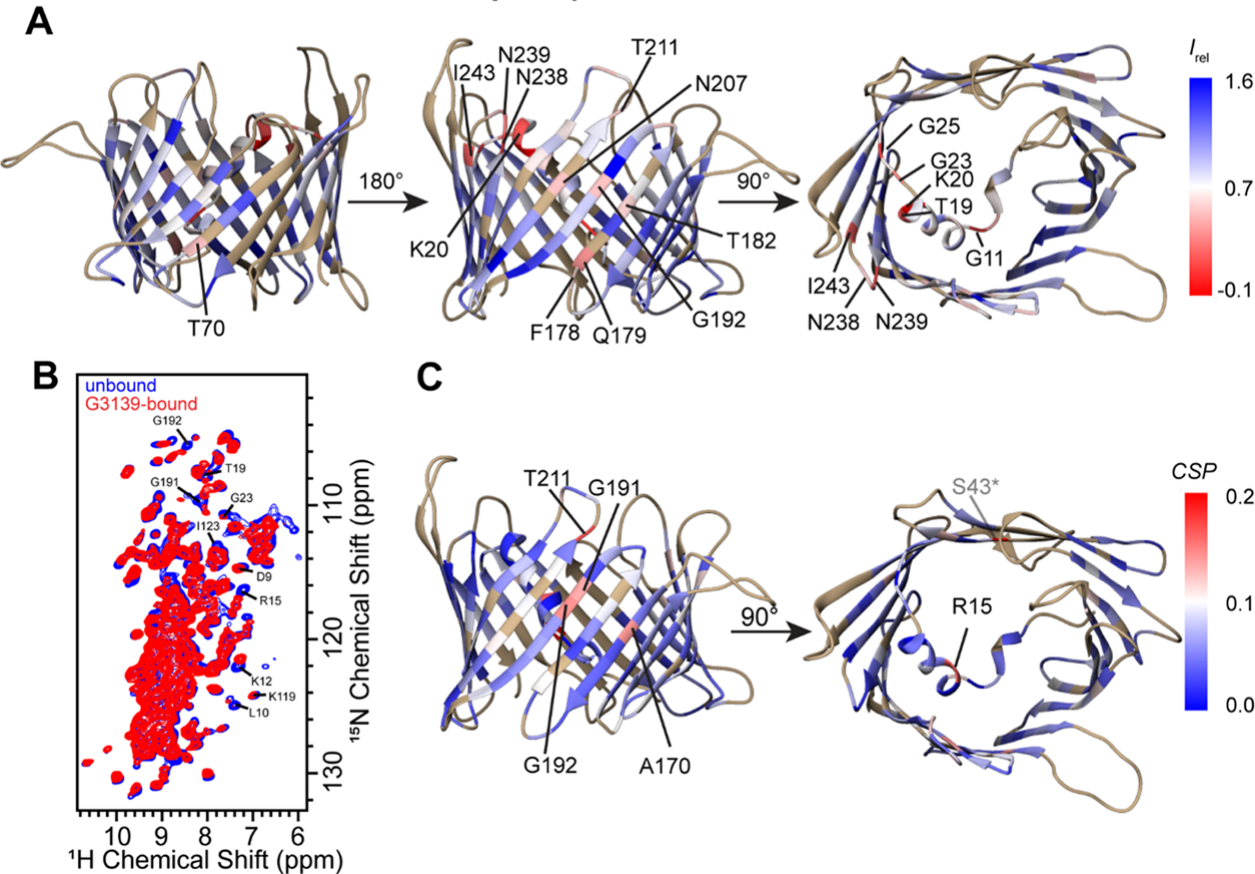
Binding of G3139 to hVDAC1(E73V/C127A/C232S). (A) Intensity changes occurring upon binding are plotted onto the lowest-energy structure of hVDAC1(E73V/C127A/C232S) in lipid bilayers with the Chimera software’s “Render by attribute” option. Relative intensities were calculated from the intensity of peaks in the (H)CANH spectra (*I*_rel_ = *I*_G3139-bound_/*I*_unbound_). If no peak could be identified, a peak was placed at the expected peak position. In the color scheme, blue corresponds to the highest relative intensity (1.5) and red corresponds to minimal relative intensity (−0.1). (B) Overlay of (H)NH spectra of the unbound state (blue) and G3139-bound state (red). (C) Chemical shift perturbations (CSP) mapped onto the lipid bilayer structure of hVDAC1(E73V/C127A/C232S). Blue corresponds to no perturbation, and red shows perturbed residues (maximal observed perturbation was 0.2 ppm). Residue S43 (marked with an asterisk) shows a large perturbation; however, the assignment for this residue in the bound state was uncertain.

The 18-mer phosphorothioate oligonucleotide G3139 (TCTCCCAGCGTGCGCCAT, the individual nucleotides linked by thiophosphate instead of phosphate bonds) has been shown to selectively block the VDAC channel, and although the mechanism is unclear, a partial entry into the pore was suggested.^53,92^ We incubated 2D crystals of hVDAC1(E73V/C127A/C232S) with G3139 solution at a 1:1.5 protein/ligand molar ratio and determined chemical shift perturbations, as well as intensity changes (Figure 6) by measuring an (H)CANH spectrum, as well as the linking (H)(CO)CA(CO)NH spectrum to confirm assignments.

No chemical shift perturbations >0.2 ppm (calculated from H^N^, ^H^N, and Cα shifts; see the Experimental Section) were observed for residues that could be unambiguously identified, however chemical shift perturbations larger than 0.1 ppm were identified in strands β13 (G191, G192) and β14 (T211) (Figure 6C). Even more striking was the fact that several residues disappeared from the (H)CANH spectrum, shown in Figure 6A,B. Residues whose intensities were most affected were G11, T19, and K20 in the α-helix; G23 and G25 in the kink between the helix and the barrel; and residues N238, N239, and I243, close to/in the loop between strands β16 and β17, on the cytosolic side of the channel.^93^

A significant decrease in peak intensities was also observed in strands β12 (F178, Q179, T182), β13 (G192), and β14 (N207). With the exception of T70, and S43, a residue whose assignment was ambiguous in the G3139-bound state, residues most affected are on the side of the barrel contacting the helix, suggesting a binding site at the helix and the neighboring, C-terminal strands of the barrel. This binding site overlaps very well with the interaction sites identified for other nucleotides (ATP,^94^ GTP, uridine triphosphate (UTP),^95^ reduced β-nicotinamide adenine dinucleotide (β-NADH)^19^).

## Discussion

### Comprehensive Sequential Assignment of hVDAC1 in a Lipid Bilayer

Membrane proteins of ∼30 kDa and above are challenging systems to investigate by MAS NMR. Both solid-state NMR of aligned samples as well as MAS NMR have been applied to the determination of an increasing number of membrane protein structures in lipid bilayer membranes.^96−98^ To date, using only MAS NMR data and lipid bilayer samples, only two β-barrel membrane protein structures have been reported.^44,99^

In previous solution NMR studies of hVDAC1, relatively complete assignments have been achieved for both wild-type hVDAC1^94^ and E73V mutants.^19^ Recently, Böhm et al. increased assignments to 88% for wild-type VDAC and to 91% for the E73V mutant in LDAO detergent micelles.^19^ They found that with the exception of some loop residues (broadened out due to exchange), all residues in the barrel could be confidently assigned (Figure S14). The assignment of the N-terminal region still remains somewhat problematic, however, as here only 12 out of the 15 helical residues (T6-K20) could be assigned, an indication of elevated exchange in this region in micelles.

The bottleneck in the MAS NMR assignment process is the usually insufficient sample quality resulting in severe overlap for large proteins. This was previously also the case for hVDAC1, where DMPC liposome samples only allowed assignment of most of the α-helix (up to V17), with the exception of a few barrel residues that could clearly be identified with selective labeling.^32^ 2D crystalline preparations^35^ in DMPC lipids for the MAS NMR investigation of hVDAC1 were a real breakthrough, as these samples gave spectra of exceptional resolution.^34^ With a ^13^C-detected assignment strategy on at least four selectively labeled 2D crystalline samples, Eddy et al. could assign not only the entire N-terminus but also stretches from seven different β-strands, a total of 88 residues (31% of all residues)^33^ (Figure S14).

Using the same 2D crystalline sample preparation on u-[^2^H,^13^C,^15^N]-hVDAC1 (the E73V/C127A/C232S mutations were introduced to improve sample stability and spectral quality), and following a proton-detected strategy for assignment,^39,41,42^ we assigned a total of 194 residues (69% of the protein) from a single sample (an Hα-labeled sample was used only to confirm assignments and obtain Hα assignments, however, no new residues were assigned based on this sample). This is comparable to the assignment completeness recently achieved in membrane proteins of similar size in lipid bilayers: 60% in the 34 kDa outer membrane protein G (OmpG) using a total of 10 amino-acid-type selective labeling schemes,^44^ and 84% in the 24 kDa membrane protein AlkL.^99^ Using only automated assignments, 58% of the protein could be assigned. It is even comparable to the 82% achieved for the 42.5 kDa maltose-binding protein (MBP) in a microcrystalline state that is particularly suitable for MAS NMR.^73^

Similar to previous MAS NMR studies, the N-terminal helix could be assigned confidently, although the first two residues assigned by Eddy et al. are missing, due to prolines breaking the backbone walk in the amide proton detection spectra and leaving an isolated stretch of only three residues. Significantly, residues from all 19 β-strands could be assigned, giving a comprehensive picture of the β-barrel in a lipid bilayer. As discussed previously by Eddy et al.,^33^ although loops often disappear from MAS NMR spectra due to their mobility, 2D lipid crystalline samples tend to introduce rigidity to these regions through the tight packing of the molecules. Indeed, we were able to assign residues from a total of 8 loops in the barrel (Figure S14).

Comparison of ^13^C-detected MAS NMR assignments given by Eddy et al. for wild-type hVDAC1^33^ and our proton-detected assignments of hVDAC1(E73V/C127A/C232S), both in a DMPC lipid bilayer, show discrepancies larger than the deuterium isotope effect (ca. 0.5–1 ppm on ^13^C and ca. 1–1.5 ppm on ^15^N)^100^ for 26 residues (chemical shift perturbation >2 ppm on HN or >1 ppm on Cα) (Figure S14). These residues do not cluster in any particular region but are scattered evenly across the barrel. This is quite surprising, as with the exception of strands around the E73V mutation site (residues ca. 40–100), no major chemical shift perturbations were observed in micelles when comparing the wild-type and the E73V mutant of the protein.^19^ Large heteronuclear (especially ^13^C) chemical shift perturbations are also not expected upon slight changes in experimental conditions such as temperature or buffer composition. A possible explanation is that in a 2D crystal, where the molecules are tightly packed against each other, the E73V mutation could also affect other regions of the neighboring β-barrels. Of course, another possible reason for chemical shift differences could be occasional misassignment. Since all perturbed residues could be assigned in the highly unambiguous 4D (H)CACONH and (H)(CO)CACONH spectra, it is unlikely that these residues were misassigned in the proton-detected approach.

### Structure of hVDAC1 in Lipid Bilayers

The structure of hVDAC1 has long been controversial. While all structural studies in detergents (micelles and bicelles)^16−20^ showed a 19-stranded β-barrel forming the bulk of the protein, these were in contrast to the structure based on functional studies:^23,101^ a barrel consisting of 13 β-strands and an α-helix.

β-barrel topologies are especially amenable to deuteration and back-exchange. Indeed, although other samples were necessary for maximizing assignments and ^1^H–^1^H contacts, both lipid bilayer structures of β-barrel membrane proteins available to date^25,44^ relied on this approach. Using a single sample to obtain ^1^H–^1^H distances, we were able to unambiguously determine hVDAC1’s topology in a lipid bilayer to be the 19-stranded β-barrel and an N-terminal α-helix, identical to the topology observed by other structural studies. While the N-terminus clearly showed the characteristic contact pattern of α-helices for residues between T6 and K20 (Figure S11), it showed a breaking point at G11, a residue showing no contacts to neighboring residues. This allows us to conclude that the structure of the N-terminus in lipid bilayers displays a kink between the two helical segments, also observed around G11 in several detergent structures,^18−20^ as well as indicated by predicted torsion angles in a lipid bilayer.^32,33^

We further confirmed the topology of the protein, as determined from the HN(H)(H)NH experiment and spin labeling by extensive torsion angle predictions based on resonance assignment and by residue-specifically mapping the protein’s environment through *z*–*z* mixing experiments.^67^

We found that the chemical shift-based prediction of torsion angles agrees well with previous structures obtained in detergents (Figure S15). Major differences (Δϕ or Δψ > 100°) were found almost exclusively in loops (Figure S16). The only exceptions to this were residues S44, G45, and G56 in one cluster. Two of these residues lack Cβ-s, making the predictions less reliable. They are also in the more mobile N-terminal β-strands, which makes it possible that they sample different conformations.

Residue-specific mapping of water and lipid proximities to the protein backbone^67^ also confirmed the 19-stranded barrel topology and contrasted starkly with the structure suggested by Colombini^101^ (Figure S17). In the latter, many residues shown to be in contact with aliphatic lipid chains would be located in loops (especially between β9 and β10). The helix, although shown by NMR to be completely exposed to water, and with no lipid contacts observed, is embedded in the membrane in Colombini’s model.

Although the chemical shift perturbations in the strands along the helix-barrel contact sites (Figure 1) indicate some minor changes in the interaction of the helix and the barrel when comparing assignments from micelles and 2D crystals, the MTSL labeling experiments and helix-barrel contacts observed in previous MAS NMR studies in lipids^32,80^ confirmed the overall orientation of the helix shown in detergent structures.

The backbone RMSD of the MAS NMR structure for structured regions is 2.2 Å, the predicted resolution being 2.7 Å.^89^ This, although comparable to the initial detergent structure of the protein,^16^ is lower than the resolution of previously reported high-resolution structures^18,19^ (ca. 1.5–2 Å). This is due to a lack of long-range restraints in our approach (due to sparse protonation), and could be further improved by full protonation at MAS frequencies >100 kHz and extensive spin labeling, which could also be used to define the barrel’s ellipticity. The RMSDs between the MAS structure and previously published VDAC structures are shown in Table S5. We find that the MAS NMR structure agrees within the ensemble RMSD with almost all other structures.

A slight ellipticity of the channel had been previously observed in hVDAC1 and mouse VDAC (mVDAC) (ε = 0.1),^17,20^ and the high-resolution structure of hVDAC1(E73V) showed an even more pronounced elliptical deformation (ε =0.2).^18,77^ The possibility was raised that this stems from the pressure exerted by the micelles onto the channel (Laplace pressure^102^), and in the case of the E73V mutant, the mutation stabilizes a certain barrel conformation. However, since an AFM study on yeast revealed highly elliptical channels (ε = 0.3) under native conditions in the mitochondrial outer membrane,^7^ it is possible that the channel is naturally noncircular (while the Laplace pressure could deform the channel in micelles, in a lipid bilayer the pressure could be coming from the tight packing of the neighboring molecules). Determining the exact ellipticity of VDAC in a lipid bilayer would, however, be extremely challenging, if at all possible by MAS NMR due to the lack of highly accurate long-distance restraints.^103^

### hVDAC1’s Mobility and Gating Behavior

hVDAC1’s voltage gating behavior is one of the channel’s most characteristic properties.^104^ A multitude of electrophysiology studies have shown the channel to be in a high-conductance, anion-selective open state in the absence of applied voltage, while under voltage, a drop in conductance and a change of selectivity can be observed.^5^ There is increasing evidence that channel gating is a highly dynamic process, with multiple open and closed substates. Both the barrel and the helix, located inside the pore in an ideal position to regulate conductance, have been suggested to partake in voltage gating.^19,20,46−48^

Peak intensities in MAS NMR are excellent indicators of dynamics, as molecular motion can manifest as weaker dipolar coupling strength, resulting in a decrease of signal intensity.^85^ In hVDAC1(E73V/C127A/C232S) (3m) (implementing the channel’s open state), we observed increased mobility in the N-terminal β-strands β1–4, as well as in the adjoining C-terminal strands β18 and β19 (peaks in β10 were also of lower intensity, however, the surrounding strands all showed strong signals). This observation agrees well with the significant peak broadening observed in detergents in the four N-terminal β-strands, as well as β16–19 in wild-type hVDAC1, and to some extent in hVDAC1(E73V).^48^ The higher intensity of peaks in β5–17, the strands adjacent to the α-helix, indicates a stabilizing effect of the helix on the barrel wall. Indeed, Schneider et al. had observed destabilization of the barrel upon truncation of the helix in ΔN(1–20)-hVDAC1 in liposomes.^32^ Compared to the rest of the barrel, the average intensity of residues in strands β1–4, 18, and 19 was roughly 65%, while the N-terminal α1-helix and α2-helix residues up until and including V17 were 20% more intense than β5–17. Interestingly, V143 in β9 (which interacts with V17 by side-chain interactions) also displayed a high intensity. Methyl group dynamics measured in LDAO micelle conditions led to different conclusions since increased mobility was detected at L10 (α1-helix), V17 (α2-helix), and also V143 (β9).^19^ While it is conceivable that the backbone is rigid as seen from the liposome experiments and the side chains adopt different rotameric states as seen from the ILV experiments of ref (19), this difference could also be due to different environments, namely, lipid bilayers versus micelles. The latter view is consistent with the observed chemical shift perturbations between lipid and detergent assignments (Figure 1A) for residues close to helix-barrel contacts including for V143.

For the closed-state quintuple (5m) (G21V/G23V/E73V/C127A/C232S) mutant in 2D lipid crystals, we found a strong increase of dynamics especially in the helix (most apparent in the α2 segment), as well as most of the barrel: in β1 as well as the adjoining C-terminal part, β6–19 (Figure 4A,B). This is in agreement with cross-linking experiments by Böhm et al.^19^ suggesting that dynamics of the α2 helix are responsible for voltage gating. This also fits well with the previous observation in liposomes that the barrel is mobilized upon truncation of the N-terminal helix in Δ(1–20)-hVDAC1, resulting in channel closure,^32^ and the molecular dynamics (MD) simulation results that large-scale deformations are needed to reproduce channel selectivity.^46^ The region β6–19 overlaps remarkably well with strands β5–19 shown in MD simulations in a DMPC bilayer to be stabilized by interaction with the helix.^46^ This raises the possibility of an at least partial dissociation of the helix (especially the α2 segment) from the barrel wall upon channel closure. The less pronounced increase in dynamics in the α1 segment and in the strands with the α1 segment running in front of them (β7–12) (Figure 4A,B), as well as an electrophysiology study showing the channel to gate normally even in the presence of an L10C-A170C cross-link^49^ suggest that the N-terminal α1 segment does not dissociate from the barrel wall.

The separate motions of the two helical segments upon voltage gating could also explain the conservation of the G11 residue among human VDAC isoforms.^105^ Since a hinge-like role^106,107^ has frequently been attributed to glycines due to their conformational flexibility, G11 could impart the possibility for the α1 and α2 helical segments to move independently.

The role of barrel mobility in channel closure is further underlined by the effect of cholesterol binding. Cholesterol binding plays a role in VDAC’s membrane insertion and also increases the probability of the channel’s open state.^52^ Our data provide a link between the channel’s electrophysiology behavior and barrel dynamics by showing that the barrel is stabilized in comparison to the helix in the presence of cholesterol (Figure 5).

A new landing site for the α-helix may also be involved in the gating mechanism. Broader lines and overall lower spectral quality of the MTSL-labeled quintuple mutant (Figure S18) could be a result of the helix sampling a large number of conformations, bringing the spin label into close proximity with residues throughout the barrel. Although the original spectrum of the MTSL-labeled quintuple mutant could not be fully recovered after reduction of the label with ascorbic acid, increasing the possibility of misfolding of the MTSL-labeled mutant, the reappearance of residues D230 and G244 in the spectrum allows us to hypothesize a second landing site for the helix at the hydrophobic patch around residues L242 and L262, both of which point into the water-filled pore.

### Comparison of Blockage Mechanisms

Although an important player in apoptosis, cancer, and neurodegeneration, VDAC’s druggability is problematic due to a lack of specific binding partners.^108^ Since the Bcl-2 antisense oligonucleotide G3139 is a promising drug candidate shown to greatly reduce channel conductance by directly binding VDAC1,^53^ we investigated the mechanism through which it inhibits hVDAC1’s normal function. We did not find changes of peak intensities in complete regions within the protein, which indicates that the mechanism of channel closure is different from the observation in the closed-state mutant (G21V/G23V/E73V/C127A/C232S), where channel closure is accompanied by mobilization of a large portion of the barrel, as well as part of the helix. Rather a decrease in intensity for individual residues in β16 and β17, as well as β12–14, in the α2 helix, as well as in the linker between the helix and the barrel was observed (Figure 6A), a signature of intermediate exchange occurring upon ligand binding.^109^ These data, taken together with the fact that the effect of the oligonucleotide is length-dependent, and upon investigating both the full-length and truncated versions of G3139, only the longest ones (14–16, and 18-mers) were found to cause significant losses of channel conductance,^53^ suggest that G3139 acts by sterically blocking the channel.

The dinucleotide β-NADH was recently shown by solution NMR and MD simulations to block the channel by steric occlusion by binding to VDAC in the same pocket at β16 and β17^19^ that we identified as the binding site of G3139. Moreover, the nucleoside triphosphate ATP has been shown to possess a binding site encompassing the α-helix, the linker between helix and barrel, and strands β12–19,^94^ which overlaps with G3139’s binding site. Other nucleotides, such as GTP and UTP have also been shown to share this site.^95^ These results indicate a common mechanism of action for nucleotide binding to VDAC. Similar steric occlusion as a blockage mechanism for the channel was also suggested for hexokinase-I and the inorganic polycationic dye ruthenium red (RuR).^110^

## Concluding Remarks

We have performed a comprehensive study of the human voltage-dependent anion channel 1’s (hVDAC1’s) structure and interactions in a lipid bilayer. Using a 2D crystalline sample giving spectra of exceptional resolution, we obtained extensive assignments for the membrane-bound channel, allowing us to determine the protein’s topology in a DMPC lipid bilayer and to calculate a structural model with a 2.2 Å RMSD. We found the topology of the protein to be the same as previously identified in micelles: an N-terminal α-helix broken into two segments by G11, and a 19-stranded β-barrel, showing a parallel orientation of strands at β1 and β19. We could identify elevated dynamics in the N-terminal β-strands β1–4, as well as in the adjacent C-terminal strands β18–19, as was also observed in micelles. We, however, found both segments of the α-helix to be exceptionally rigid in the open state, in contrast to the case in micelles.

The closed state of hVDAC1, implemented by introducing the two additional G21V/G23V mutations to the linker between the helix and the barrel, showed an increase in dynamics in the N-terminal helix, particularly the α2 segment, as well as in the β-strands previously stabilized by the helix. This provides further evidence for the closed state of hVDAC1 being highly dynamic, with both α2 helix displacement and barrel elliptic deformation participating in voltage gating. We showed that cholesterol reduces barrel motions in the open state. Furthermore, we showed the binding site of the specific binding partner and cancer drug candidate G3139 to coincide with nucleotide-binding sites observed in micelles.

## Supporting Information Available

Additional images, spectra, and tables (PDF)

## Abbreviations

MAS NMR: magic-angle spinning NMR spectroscopy
hVDAC1: human voltage-dependent anion channel 1
AFM: atomic force microscopy
ATP: adenosine triphosphate
ADP: adenosine diphosphate
MTSL: *S*-(1-oxyl-2,2,5,5-tetramethyl-2,5-dihydro-1*H*-pyrrol-3-yl)methyl methanesulfonothioate
IPTG: isopropyl β-d-1-thiogalactopyranoside
PMSF: phenylmethylsulfonyl fluoride
β-NADH: reduced β-nicotinamide adenine dinucleotide
RuR: ruthenium red
*n*-octyl-PEO: *n*-octylpolyoxyethylene
MES: 2-(*N*-morpholine)ethanesulfonic acid
DTT: dithiothreitol
GTP: guanosine triphosphate
UTP: uridine triphosphate
DMPC: 1,2-dimyristoyl-*sn*-glycero-3-phospho-choline
DPhPC: 1,2-diphytanoyl-*sn*-glycero-3-phosphocholine
α-PET: α proton exchange by transamination
