# Supplementary material for: Structure and Gating Behavior of the Human Integral Membrane Protein VDAC1 in a Lipid Bilayer
Source: J Am Chem Soc. 2022 Feb 14;144(7):2953–67. doi: 10.1021/jacs.1c09848 (PMC8874904; doi:10.1021/jacs.1c09848)
Supplement: Supplementary file 1 — ja1c09848_si_001.pdf [file ja1c09848_si_001.pdf]

Supporting Information

Structure and Gating Behavior of the Human Integral Membrane Protein VDAC1 in a Lipid Bilayer

Eszter E. Najbauer[1], Kumar Tekwani Movellan[1], Karin Giller[1], Roland Benz[2], Stefan Becker[1], Christian Griesinger[1], Loren B. Andreas[1]*

[1]Department of NMR-based Structural Biology, Max Planck Institute for Multidisciplinary Sciences, Am Faßberg 11, 37077 Göttingen, Germany

[2]Life Sciences and Chemistry, Jacobs University of Bremen, Campus Ring 1, 28759 Bremen, Germany

Table S1: Composition of M9 medium supplemented with trace elements and isotope labels for bacterial cultures

Components	Final conc.
Na_2HPO_4	47.76 mM
KH_2PO_4	22 mM
NaCl	8.55 mM
$MgSO_4$	2 mM
$CaCl_2$	0.1 mM
$FeSO_4$	0.216 mM
$ZnSO_4$	24.34 µM
$MnCl_2$	58.1 µM
H_3BO_3	3.24 µM
$(NH_4)_6Mo_7O_{24}$	2.15 µM
$CoCl_2$	33.6 µM
$CuCl_2$	17.6 µM
EDTA	0.15 mM
Thiamine hydrochloride	89 µM
$^{15}NH_4Cl$	1 g/L
D-Glucose U-$^{13}C_6$	4.0 g/L

Table S2: Experiments and experimental parameters. Two different HN(H)(H)NH experiments were recorded, one with non-uniform, the other with uniform sampling, the latter's parameters appearing in parentheses whenever they are not identical.

**Spectrum**	**(H)NH**	**(H)CANH**	**(H)(CO)CA(CO)NH**	**(H)CONH**	**(H)CO(CA)NH**
Scans / point	48	36	28	16	64
Experimental time / hours	14	151	117.5	34.8	139.8
Field / MHz	800	800	800	800	800
Spinning frequency / Hz	55000	55000	55000	55000	55000
Sweep width (t_1) / ppm	120.0 (^{15}N)	33.2 (^{15}N)	33.2 (^{15}N)	40.0 (^{15}N)	40.0 (^{15}N)
Max indirect evolution (t_1) / ms	52.6 (^{15}N)	21.9 (^{15}N)	21.9 (^{15}N)	22.2 (^{15}N)	22.2 (^{15}N)
Sweep width (t_2) / ppm	30.0 (^{1}H)	32.7 (^{13}C)	32.7 (^{13}C)	16.0 (^{13}C)	16.0 (^{13}C)
Max indirect evolution (t_2) / ms	54.9 (^{1}H)	9.7 (^{13}C)	9.7 (^{13}C)	22.1 (^{13}C)	22.1 (^{13}C)
Sweep width (t_3) / ppm		30.0 (^{1}H)	30.0 (^{1}H)	30.0 (^{1}H)	30.0 (^{1}H)
Max indirect evolution (t_3) / ms		21.3 (H)	21.3 (H)	21.3 (H)	21.3 (H)
Transfer I	HN (dipolar)	HCA (dipolar)	HCO (dipolar)	HCO (dipolar)	HCO (dipolar)
^{1}H field / kHz	90	90	90	87.83	91.3
X field / kHz	39.7	38.2	38.22	37.235	41.15
Shape	80 - 100% linear (^{1}H)	80 - 100% linear (^{1}H)	80 - 100% linear (^{1}H)	80 - 100% linear (^{1}H)	80 - 100% linear (^{1}H)
Carrier ^{13}C		53.7	173.3	173.8	173.8
Time / ms	1	6	6	5	5
Transfer II	NH (dipolar)	CAN (dipolar)	COCA (scalar)	CON (dipolar)	COCA (scalar)
^{1}H field / kHz	84.4				
^{13}C field / kHz		23.31		22.886	
^{15}N field / kHz	18.1	29.83		30.16	
Carrier ^{13}C		53.7	173.3 and 53.3	173.8	173.8 and 53.3

Spectrum	(H)NH	(H)CANH	(H)(CO)CA(CO)NH	(H)CONH	(H)CO(CA)NH
Shape	100-80% linear (^{1}H)	63-94% tangential (^{15}N)		63-94% tangential (^{15}N)	
Transfer II	NH (dipolar)	CAN (dipolar)	COCA (scalar)	CON (dipolar)	COCA (scalar)
Time / ms	0.6	8	9.1 (1st step)	12	7.6 (1st step) and 6.2 (second step)
Transfer III		NH (dipolar)	CON (dipolar)	NH (dipolar)	CAN (dipolar)
^{1}H field / kHz		84.43		84.8	
^{13}C field / kHz			23.31		23.36
^{15}N field / kHz		39.71	29.83	39.707	30.83
Carrier ^{13}C			173.3		53.3
Shape		100 - 80% linear (^{1}H)	63-94% tangential (^{15}N)	100 - 80% linear (^{1}H)	63-94% tangential (^{15}N)
Time / ms		0.6	8	0.6	10
Transfer IV			NH (dipolar)		NH (dipolar)
^{1}H field / kHz			83.43		86.5
^{15}N field / kHz			39.71		39.71
Shape			100 - 80% linear (^{1}H)		100 - 80% linear (^{1}H)
Time / ms			0.6		0.6

Spectrum	(H)(CA)CB(CA)NH	(H)(CA)CB(CA)(CO)NH	(H)N(CA)(CO)NH	(H)N(CO)(CA)NH
Scans / point	8	14	32	28
Experimental time / hours	163.8	286.7	157.2	137.6
Field / MHz	950	950	800	800
Spinning frequency / Hz	90909	90909	55000	55000
Sweep width (t_1) / ppm	40.0 (^{15}N)	40.0 (^{15}N)	36.0 (^{15}N)	36.0 (^{15}N)
Max indirect evolution (t_1) / ms	20.8 (^{15}N)	20.8 (^{15}N)	23.0 (^{15}N)	23.0 (^{15}N)
Sweep width (t_2) / ppm	80.5 (^{13}C)	80.5 (^{13}C)	36.0 (^{15}N)	36.0 (^{15}N)

Spectrum	(H)(CA)CB(CA)NH	(H)(CA)CB(CA)(CO)NH	(H)N(CA)(CO)NH	(H)N(CO)(CA)NH
Max indirect evolution (t_2) / ms	10.0 (^{13}C)	10.0 (^{13}C)	22.6 (^{15}N)	22.6 (^{15}N)
Sweep width (t_3) / ppm	52.6 (^{1}H)	52.6 (^{1}H)	30.0 (^{1}H)	30.1 (^{1}H)
Max indirect evolution (t_3) / ms	41.0 (^{1}H)	41.0 (^{1}H)	21.3 (^{1}H)	21.3 (^{1}H)
Transfer I	HCA (dipolar)	HCA (dipolar)	HN (dipolar)	HN (dipolar)
^{1}H field / kHz	72.9	72.9	84.91	85.18
X field / kHz	15.3	15.3	37.3	37.3
Shape	85 - 100% linear (^{1}H)	85 - 100% linear (^{1}H)	80 - 100% linear (^{1}H)	80 - 100% linear (^{1}H)
Carrier ^{13}C	53.7	53.7		
Time / ms	4	4	1	1
Transfer II	CACB (scalar, out-and-back)	CACB (scalar, out-and-back)	NCA (dipolar)	NCO(dipolar)
^{13}C field / kHz			23.67	25.4
^{15}N field / kHz			28.02	27.89
Carrier ^{13}C	39.7	39.7	53.3	173.3
Shape			63-94% tangential (^{15}N)	63-94% tangential (^{15}N)
Time / ms	10.4	10.4	13	16
Transfer III	CAN (dipolar)	CACO (scalar)	CACO (scalar)	COCA (scalar)
^{1}H field / kHz				
^{13}C field / kHz	58.8			
^{15}N field / kHz	41.85			
Carrier ^{13}C	30.1	173.3 and 53.3	53.3 and 173.3	173.3 and 53.3
Shape	76-96% tangential (^{15}N)			
Time / ms	19	5.7 ms (1st, simultaneous with CB), 8.2 2nd transfer	7.5 (1st step) and 6.7 (2nd step)	7.5 (1st step) 6.7 (2nd step)
Transfer IV	NH (dipolar)	CON (dipolar)	CON (dipolar)	CAN (dipolar)
^{1}H field / kHz	110.1			
^{13}C field / kHz		60.4	24.21	25.07

Spectrum	(H)(CA)CB(CA)NH	(H)(CA)CB(CA)(CO)NH	(H)N(CA)(CO)NH	(H)N(CO)(CA)NH
^{15}N field / kHz	35	32.3	35.71	35.87
Transfer IV	NH (dipolar)	CON (dipolar)	CON (dipolar)	CAN (dipolar)
Carrier ^{13}C		173.7	173.3	53.3
Shape	80 - 100% linear (^{1}H)	76-96% tangential (^{15}N)	63-94% tangential (^{15}N)	63-94% tangential (^{15}N)
Time / ms	0.7		12	16
Transfer V		NH (dipolar)	NH (dipolar)	NH (dipolar)
^{1}H field / kHz		110.1	80.325	82.23
^{15}N field / kHz		35	37.3	37.3
Shape		80 - 100% linear (^{1}H)	100 - 80% linear (^{1}H)	100 - 80% linear (^{1}H)
Time / ms		0.7	0.6	0.7

Spectrum	(H)COCANH	(H)(CO)CACONH	(H)COCA(N)H	HN(H)(H)NH
NUS%	2.86	3.21		7.87 (-)
Scans / point	16	6	28	16 (12)
Experimental time / hours	149.9	90.5	141.4	194 (156)
Field / MHz	800	800	800	800 (850)
Spinning frequency / Hz	55000	55000	55000	55000
Sweep width (t_1) / ppm	16.0 (^{13}CO)	34.0 (^{15}N)	16.0 (^{13}CO)	5.5 (6.0) (^{1}H)
Max indirect evolution (t_1) / ms	22.1 (^{13}CO)	22.1 (^{15}N)	22.1 (^{13}CO)	4.0 (2.5) (^{1}H)
Sweep width (t_2) / ppm	32.7 (^{13}CA)	34.0 (^{13}CA)	32.7 (^{13}CA)	33.0 (^{15}N)
Max indirect evolution (t_2) / ms	9.7 (^{13}CA)	13.0 (^{13}CA)	9.7 (^{13}CA)	22.0 (10.5) (^{15}N)
Sweep width (t_3) / ppm		15.0 (^{13}CO)	30.1 (1H)	33.0 (^{15}N)
Max indirect evolution (t_3) / ms		12.9 (^{13}CO)	21.3 (^{1}H)	22.0 (10.5) (^{15}N)

**Spectrum**	**(H)COCANH**	**(H)(CO)CACONH**	**(H)COCA(N)H**	**HN(H)(H)NH**
Sweep width (t_4) / ppm	30.1 (H)	30.0 (H)		30.0 (H)
Max indirect evolution (t_4) / ms	21.3 (H)	21.3 (H)		21.3 (H)
Transfer I	HCO (dipolar)	HCO (dipolar)	HCO (dipolar)	HN (dipolar)
^{1}H field / kHz	91.277	94.87	76.85	96
X field / kHz	41.154	38.44	41.32	39.06
Shape	80 - 100% linear (^{1}H)	80 - 100% linear (^{1}H)	80 - 100% linear (^{1}H)	80 - 100% linear (^{1}H)
Carrier ^{13}C	173.3	173.3	173.3	
Time / ms	5	6	6	1
Transfer II	COCA (scalar, SCT)	COCA (scalar, out-and-back)	COCA (scalar, SCT)	NH (dipolar)
^{1}H field / kHz				91.03
^{15}N field / kHz				39.06
Carrier ^{13}C	173.3 then 53.3	173.3 and 53.3	173.3 then 53.3	
Shape				100 - 80% linear (^{1}H)
Time / ms	7.6 (1st transfer) and 6.7 (2nd transfer)	7.5	7.2 (1st transfer) 5.4 (2nd transfer)	0.6
Transfer III	CAN (dipolar)	CON (dipolar)	CAN (dipolar)	HH (RFDR)
^{1}H field / kHz				
^{13}C field / kHz	23.36	24.38	25.2	
^{15}N field / kHz	30.83	29.83	28.76	
Carrier 13C	53.3	173.3	53.3	
Shape	63-94% tangential (N)	63-94% tangential (N)	63-94% tangential (N)	
Time / ms	10	8	8	2.3
Transfer IV	NH (dipolar)	NH (dipolar)	NH (dipolar)	HN (dipolar)
^{1}H field / kHz	86.5	88.99	76.48	96
^{15}N field / kHz	39.71	39.71	38.47	39.06

Spectrum	(H)COCANH	(H)(CO)CACONH	(H)COCA(N)H	HN(H)(H)NH
Shape	100 - 80% linear (^{1}H)	100 - 80% linear (^{1}H)	100 - 80% linear (^{1}H)	80 - 100% linear (^{1}H)
Time / ms	0.6	0.6	0.6	1
Transfer V				NH (dipolar)
^{1}H field / kHz				91.03
^{15}N field / kHz				39.06
Shape				100 - 80% linear (^{1}H)
Time / ms				0.6

Spectrum	(H)NCAH	(H)N(CO)CAH
Scans / point	12	48
Experimental time / hours	50.3	54.6
Field / MHz	800	800
Spinning frequency / Hz	55000	55000
Sweep width (t_1) / ppm	33.0 (^{15}N)	33.0 (^{15}N)
Max indirect evolution (t_1) / ms	22.1 (^{15}N)	11.6 (^{15}N)
Sweep width (t_2) / ppm	33.0 (^{13}CA)	33.0 (^{13}CA)
Max indirect evolution (t_2) / ms	9.6 (^{13}CA)	5.0 (^{13}CA)
Transfer I	HN (dipolar)	HN (dipolar)
^{1}H field / kHz	94.093	94.093
X field / kHz	38.466	38.466
Shape	80 - 100% linear (^{1}H)	80 - 100% linear (^{1}H)
Time / ms	1.4	1.4

**Spectrum**	**(H)NCAHA**	**(H)N(CO)CAHA**
Transfer II	NCA (dipolar)	NCO (dipolar)
^{15}N field / kHz	33.760	33.760
^{13}C field / kHz	21.069	21.069
Carrier ^{13}C	53.7	173.3
Shape	63-94% tangential (N)	63-94% tangential (N)
Time / ms	18	18
Transfer III	CAHA (dipolar)	COCA (scalar, SCT)
^{1}H field / kHz	87.882	
^{13}C field / kHz	42.137	
Carrier ^{13}C	53.3	173.3 then 53.3
Shape	100 - 80% linear (^{1}H)	
Time / ms	0.3	7.2 (1st transfer)<br>5.4 (2nd transfer)
Transfer IV		CAH (dipolar)
^{1}H field / kHz		87.882
^{13}C field / kHz		42.137
Carrier ^{13}C		53.3
Shape		100 - 80% linear (^{1}H)
Time / ms		0.3

Table S3: Confident assignments obtained with the FLYA algorithm. The assignment of a chemical shift value was classified as confident by the program, when for the residue the algorithm converged to the same value in 80% of the cases. A residue was counted as confidently assigned when it had at least two of its backbone atoms confidently assigned, and was part of a stretch of at least two such residues. Basic 6 experiments are defined as following: (H)CANH, (H)(CO)CA(CO)NH, (H)CONH, (H)CO(CA)NH, (H)(CA)CB(CA)NH, (H)(CA)CB(CA)(CO)NH

Peak lists used for automated assignments	Confidently assigned residues	% of protein confidently assigned
Basic 6	74	26.1%
Basic 6, (H)COCANH, (H)(CO)CA(CO)NH	150	53.0%
Basic 6, (H)COCANH, (HCO)CA(CO)NH	137	48.4%
Basic 6, (H)N(COCA)NH, (H)Nc(CACO)NH	162	57.2%
Basic 6, (H)COCANH, (HCO)CA(CO)NH, (H)N(COCA)NH, (H)N(CACO)NH	102	36.0%
Basic 6, (H)NCAH, (H)N(CO)CAH	57	20.1%
(H)COCANH, (HCO)CA(CO)NH, (HCA)CB(CA)NH, (HCA)CB(CACO)NH	163	57.6%
Basic 6, (H)NCAH, (H)N(CO)CAH, (H)COCANH, (HCO)CA(CO)NH, (H)N(COCA)NH, (H)N(CACO)cNH	161	56.9%
Basic 6, (H)COCANH, (HCO)CA(CO)NH, (H)N(COCA)NH, (H)N(CACO)NH, (H)COCA(N)H	163	57.6%

Table S4: Restraints used in structure calculation

H-bonds (from MAS-NMR data, this work): H-bonds modeled in based on contacts in the HN(H)(H)NH spectrum. Restraints were included both as lower (H-O: 1.8 Å and N-O: 2.7 Å) and upper (H-O: 2.0 Å and N-O: 3.0 Å) distances. Upper distance restraints were weighted with a factor of 5.0 to facilitate convergence. Residue pairs marked with (*) were not included as H-bonded in the upper distance restraint file, but were set to have a H^N-H^N distance of 4 Å.

T6 – D9	S13 – D16	L26 – L277	K28 – L277
K28 – L279	I27 – A47	L29 – G45	T42 – E59
S46 – T55	Y62 – F71	W64 – L69*	T70 – T86
T72 – E84	V73 – T60	I85 – F99	V87 – L97
K96 – G117	T98 – K115	D100 – K113	S101 – T83
I114 – M129	Y118 – L125	R120 – I123*	N124 – V143
G126 – A141	A127 – T116	D128 – R139	D130 – S137
G140 – M155	L142 – Y153	L144 – A151	L150 – G172
G152 – A170	Q154 – N168	F169 – V184	V171 – T182
Y173 – L180	T175 – F178*	Q179 – Y195	H181 – S193
N183 – G191	N185 – E189	F190 – W210	G192 – L208
I194 – V206	Q194 – T204	E203 – K224	A205 – A222
N207 – G220	A209 – R218	I221 – V237	A223 – A235
Y225 – F233	S232 – T248	S234 – G246	K236 – G244
L245 – A261	Y247 – L259	L257 – Q249	T258 – G278
S260 – G276			

H-bonds (modeled based on secondary structure prediction from chemical shift information): After the topology of the barrel was established based on the H-H contacts observed in the HN(H)(H)NH spectrum, additional H-bonds were modeled based on the TALOS-N secondary structure predictions. For the H-bonds below, one residue was predicted to be in extended conformation based on chemical shifts, the other based on a sequence-based prediction. H-bonds were included both as lower (H-O: 1.8 Å and N-O: 2.7 Å) and upper (H-O: 2.0 Å and N-O: 3.0 Å) distance restraints. In case of the two H-bonds in italics, both residues were predicted to be in extended conformation by TALOS-N's sequence-based prediction. Upper distance restraints were weighted with a factor of 5.0 to facilitate convergence. Residue pairs marked with (*) were not included as H-bonded in the upper distance restraint file, but were set to have a H^N-H^N distance of 4 Å.

D30 – L279	D30 – F281	K32 – F281	L31 – S43
E40 – K61	S44 – S57	G56 – T77	A112 – F131
Y146 – W149*	A209 – R218	I243 – L263	K256 – E280
L262 – K274	*T33 – F41*	*L58 – W75*	

Helix-barrel restraints from paramagnetic relaxation enhancement (hVDAC1(E73V/C127A/C232S)-A2C-MTSL). The restraints were set to have an upper limit of 10 Å.

A2 Cβ – R120 H	A2 Cβ – E121 H	A2 Cβ – H122 H	A2 Cβ – I123 H
A2 Cβ – N124 H			

Helix-barrel contacts from previous ^{13}C-detected MAS NMR data[1-2] **Upper** distance restraints were set to 6 Å, and a lower limit of 4 Å.

A14 Cβ – S193 Cβ	L10 Cγ –V143 Cβ	L10 Cγ – V143 Cα	

Restraints defining the barrel shape based on AFM data.[3] Distances between the Cα atoms of opposing residues were set to a lower limit of 27.0 Å and an upper limit of 38.0 Å. Residues one quarter of a barrel away (shown in italics) were set to a lower limit of 19.1 Å and an upper limit of 26.9 Å (distance between Cα atoms).

L29 – Y153	L29 – A170	S44 – A170	S44 – T182
L58 – T182	L58 – I194	V73 – I194	V73 – A205
E84 – A205	E84 – A223	F99 – A223	F99 – S234
K115 – V237	K115 – Y247	A127 – Y247	A127 – T258
A141 – A258	A141 – L279	Y153 – L279	*L29 – F99*
S44 – K115	*L58 – A127*	*V73 – A141*	*E84 – Y153*
F99 – A170	*K115 – T182*	*A127 – I194*	*A141 – A205*
Y153 – A223	*A170 – S234*	*T182 – Y247*	*I194 – T258*
A205 – L279	*A223 – L29*	*S234 – S44*	*Y247 – L58*
T258 – V73	*L279 – E84*		

Table S5: Backbone and heavy atom RMSD to the mean between the MAS NMR structure and previously published VDAC structures (Figure S1). In the case of NMR structures, the structure most representative of the ensemble as determined by WHAT IF[4] was used for calculation of the RMSDs. The residues used for determining the RMSDs are those in regular secondary structures in the lowest energy model of the structure reported herein: 6-9, 13-22, 27-32, 40-47, 55-63, 70-77, 83-87, 97-101, 112-118, 123-131, 137-144, 149-156, 168-174, 179-185, 189-199, 202-210, 218-225, 232-237, 243-250, 256-264, 272-280. When determining the RMSD without the helix, residues 6-9 and 13-22 were not taken into account.

Structure	Backbone RMSD (Å)	Heavy atom RMSD (Å)	Backbone RMSD (w/o helix) (Å)	Heavy atom RMSD (w/o helix) (Å)
Hiller et al, 2008[5] (2k4t)	2.16	2.53	1.61	2.05
Bayrhuber et al, 2008[6] (2jk4)	1.76	2.19	1.37	1.89
Ujwal et al, 2008[7] (3emn)	1.73	2.16	1.28	1.72
Jaremko et al, 2016[8] (5jdp)	2.55	2.88	2.33	2.65
Hosaka et al, 2017[9] (5xdn)	1.73	2.14	1.25	1.67
Martynowycz et al, 2020[10] (7kuh)	1.71	2.16	1.28	1.73
Bohm et al, 2020 (6tiq)[11]	1.64	2.09	1.46	1.93

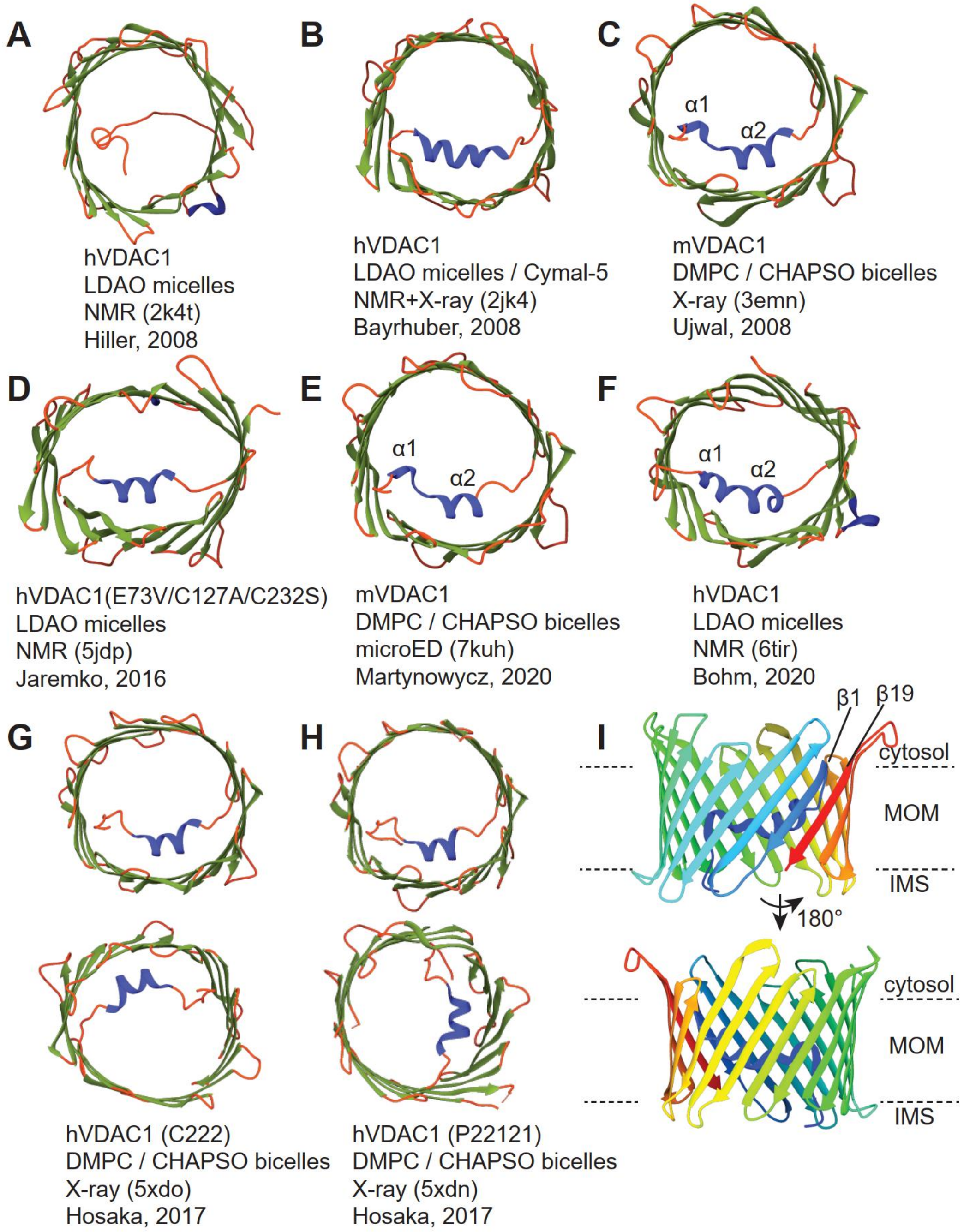

Figure S1: VDAC structure in micelles / bicelles. A) Human VDAC1 (hVDAC1) in LDAO micelles using NMR spectroscopy (pdb: 2k4t).[5] B) hVDAC1 as determined by a combination of NMR spectroscopy (LDAO detergent micelles) and X-ray crystallography (Cymal-5 detergent) (pdb. 2jk4).[6] C) Mouse VDAC1 (mVDAC1) crystallized from DMPC/CHAPSO bicelles as determined by X-ray crystallography (pdb: 3emn).[7] D) hVDAC1(E73V) in LDAO micelles as determined by NMR spectroscopy (pdb: 5jdp).[8] E) mVDAC1 crystallized from DMPC/CHAPSO bicelles determined by microcrystal electron diffraction (microED) (pdb. 7kuh).[10] F) hVDAC1 in LDAO micelles determined by NMR spectroscopy (pdb. 6tir).[11] hVDAC1 crystallized from DMPC/CHAPSO bicelles determined by X-ray crystallography in the G) C222 space group (pdb: 5xdo)[9] and in the H) P22121 space group (pdb: 5xdn).[9] I) Shows mVDAC1 (pdb: 3emn) from the side, indicating the cytosol, the mitochondrial outer membrane (MOM), and the intermembrane space. The α1 and α2 helical segments are indicated on panels C), E), and F).

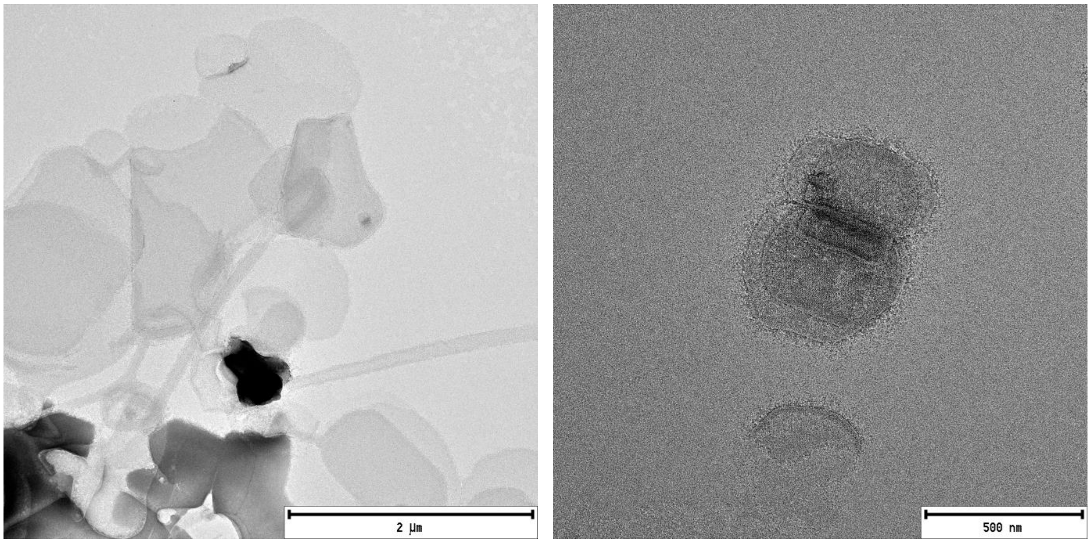

Figure S2: Electron micrographs of hVDAC1(E73V/C127A/C232S) 2D crystals. Scale bars are indicated on the bottom right of the images. The lamellar structure of the sample is clearly visible on both images, on the right, VDAC channels are visible as small black dots.

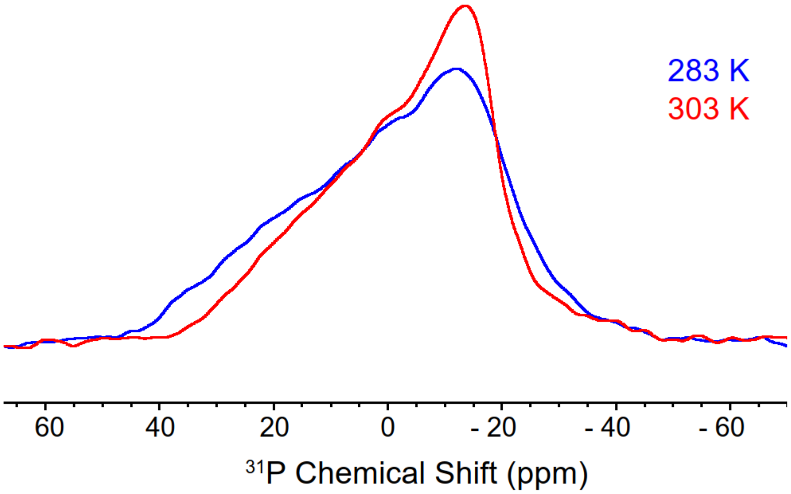

Figure S3: ^{31}P powder pattern of 2D DMPC lipid crystalline hVDAC1(E73V/C127A/C232S). Spectra were acquired on a 599 MHz spectrometer, in the absence of magic angle spinning at 283 K (blue) and 303 K (red).

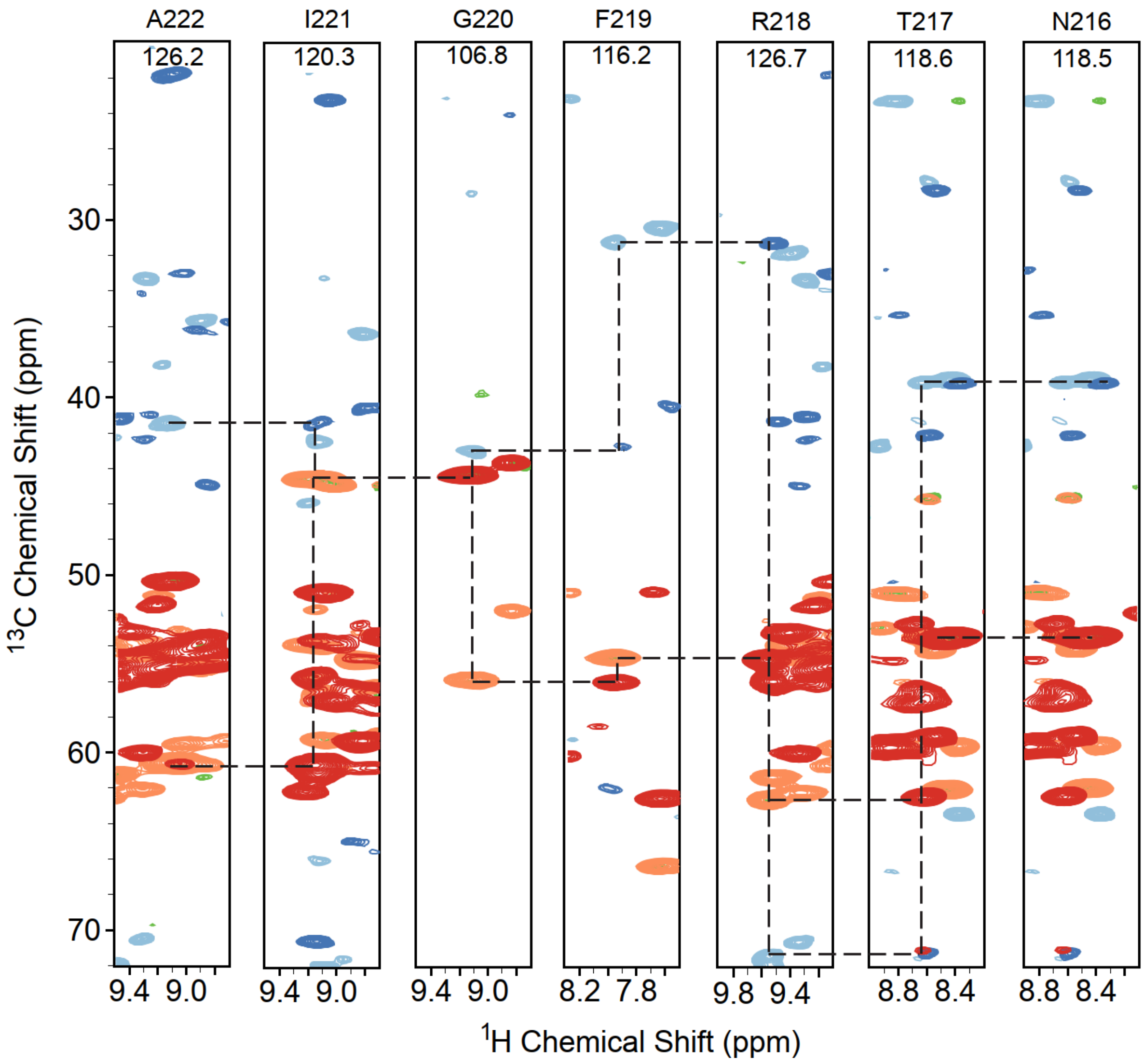

Figure S4: Backbone walk showing the proton-detected assignment strategy using (H)CANH (red), (HCO)CA(CO)NH (orange), (HCA)CB(CA)NH (dark blue), and (HCA)CB(CACO)NH (light blue) spectra. Residues shown are in β15 and the preceding loop, previously unassigned by solid state MAS NMR.

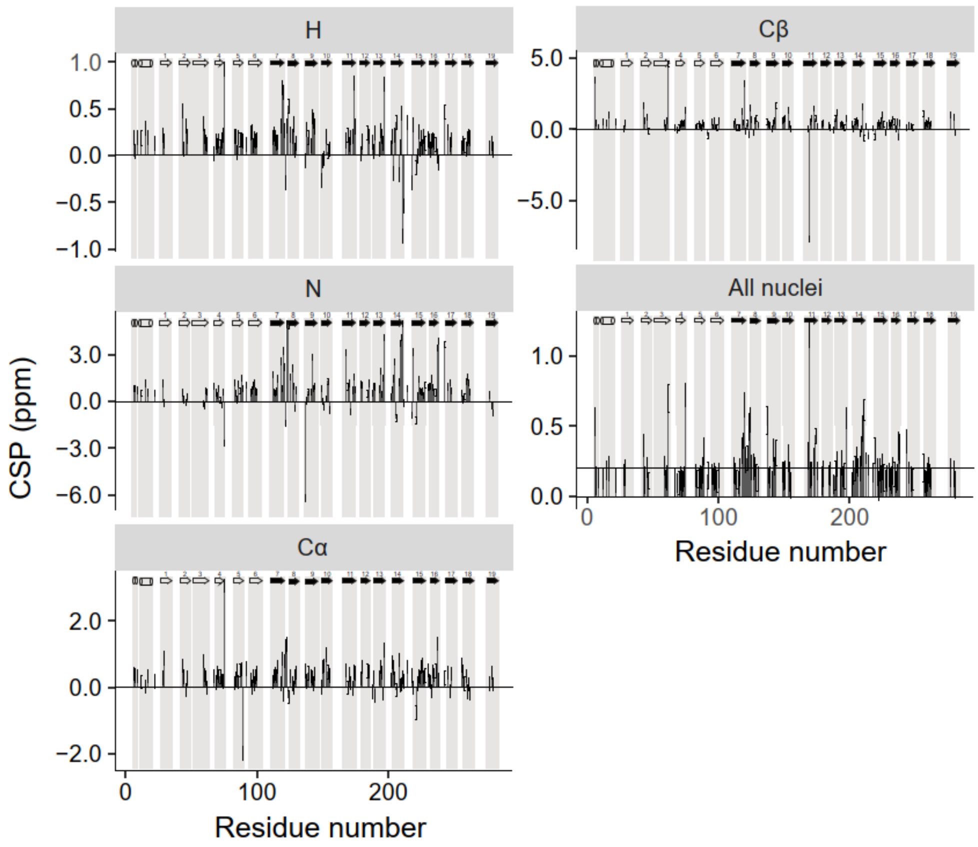

Figure S5: Chemical shift perturbations on ^{1}H, ^{15}N, $^{13}C\alpha$, $^{13}C\beta$ and their combination (as presented in the main text). The weighted chemical shift perturbations were calculated as described in the Methods section[12] with scaling factors of ^{1}H: 1, ^{15}N: 0.17, and ^{13}C: 0.3.

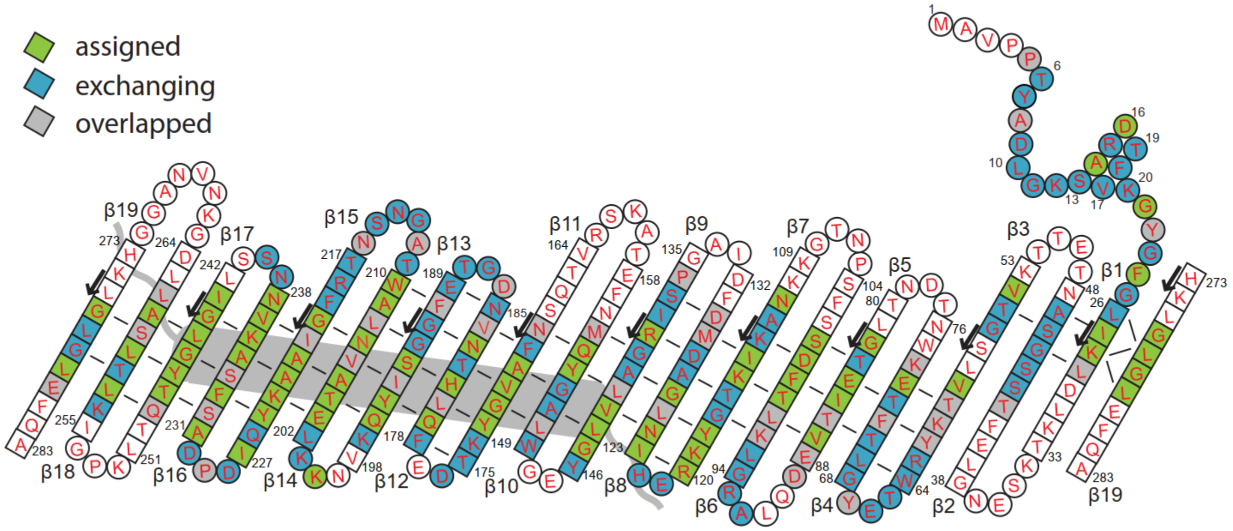

Figure S6: H/D exchange of hVDAC1(E73VC127AC232S). Residues undergoing complete exchange ($I < 3\sigma$, where σ is the noise level) in the 1 day timeframe of the experiment are shown in blue, other assigned residues are shown in green. Assigned residues where signal overlap interferes with determining exact signal intensities are shown in grey. The helix running along the barrel wall is shown in gray.

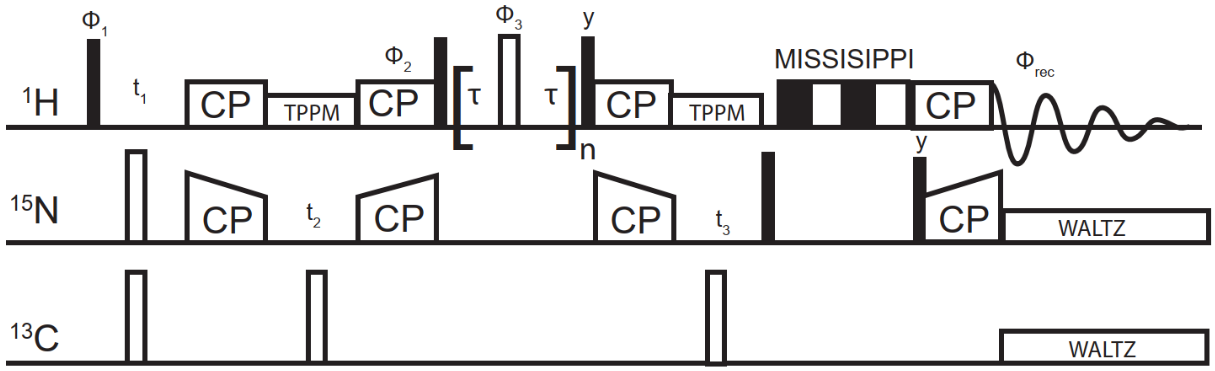

Figure S7: HN(H)(H)NH pulse sequence for acquiring spatial restraints. Full and empty rectangles denote hard excitation and inversion pulses respectively. ^{1}H-^{1}H dipolar mixing is achieved by repeating the RFDR block enclosed by brackets *n* times. The refocusing pulses in the RFDR sequence are applied in a rotor synchronized manner, and the length of one block is set to exactly one rotor period (1 / υ_{rot}). All phases were *x* unless noted otherwise. Phase cycling is as follows: $\phi_1 = y\ \text{-}y$, $\phi_2 = (y)_2(\text{-}y)_2$, $\phi_3 = (x\ y)_2(y\ x)_2$, and $\phi_{rec} = x\ \text{-}x\ \text{-}x\ x$.

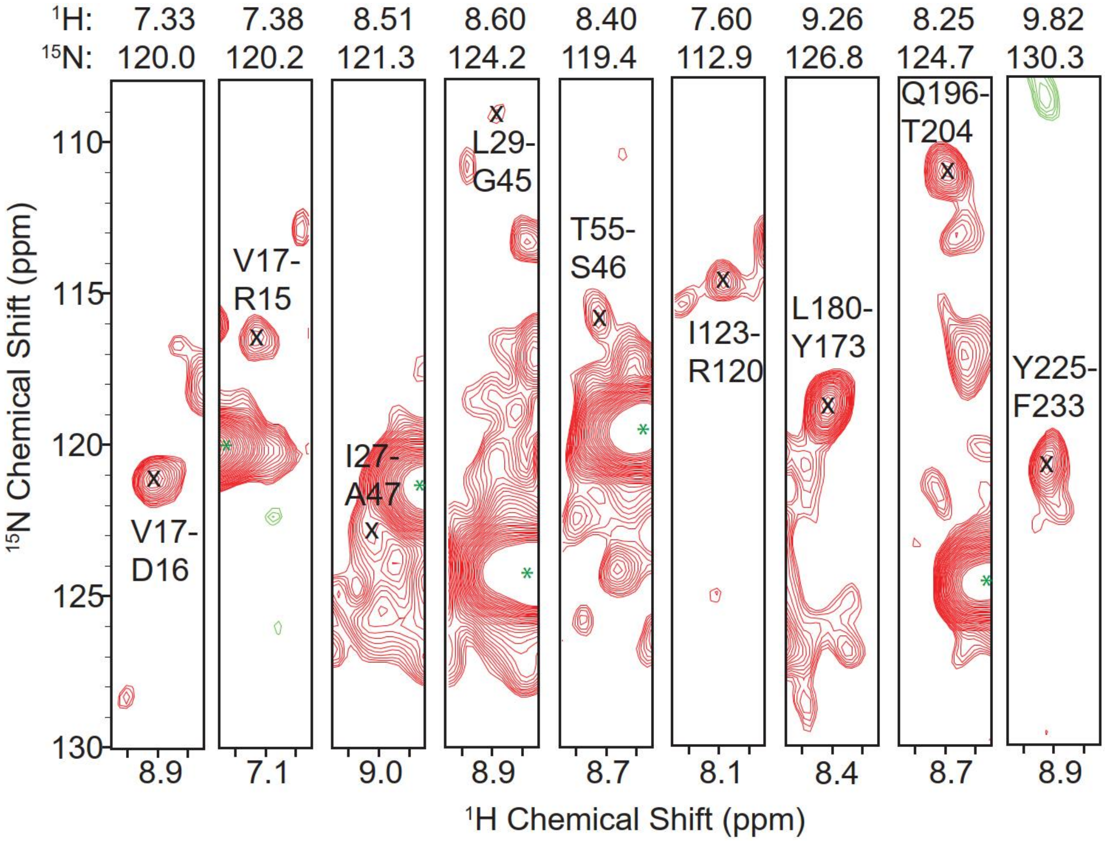

Figure S8: Strips showing interresidue contacts from the HN(H)(H)NH spectrum. All strips were selected from the uniformly sampled HN(H)(H)NH spectrum to show both helical contacts (V17-D16, V17-R15) and contacts between neighboring strands in the β-barrel (all other strips). All three contacts marked as ambiguous on Figure S7A are displayed (I27-A47, L29-G45, T55-S46). Green stars indicate diagonal peaks (this is in some cases not in the range displayed in the strip).

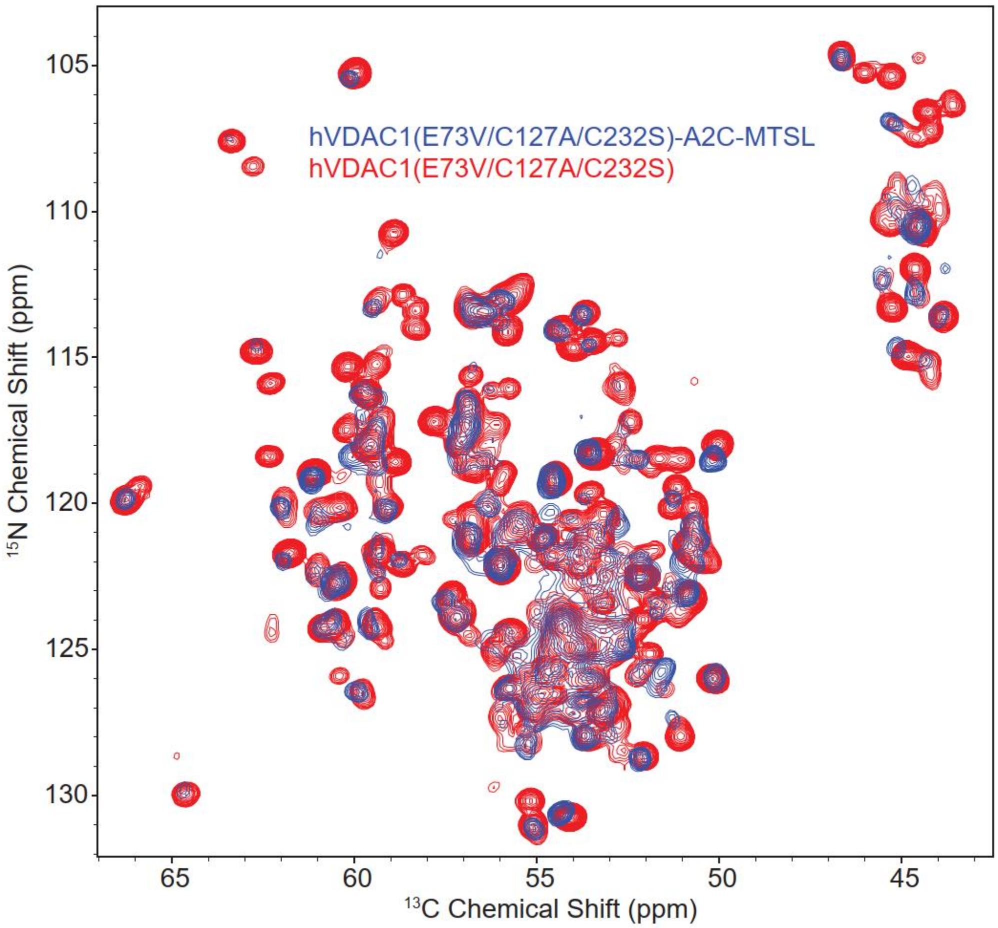

Figure S9: MTSL labeling of hVDAC1(E73V/C127A/C232S. NC projections of the (H)CANH spectra of unlabeled hVDAC1(E73V/C127A/C232S) (red) and hVDAC1(E73V/C127A/C232S)-A2C-MTSL (blue).

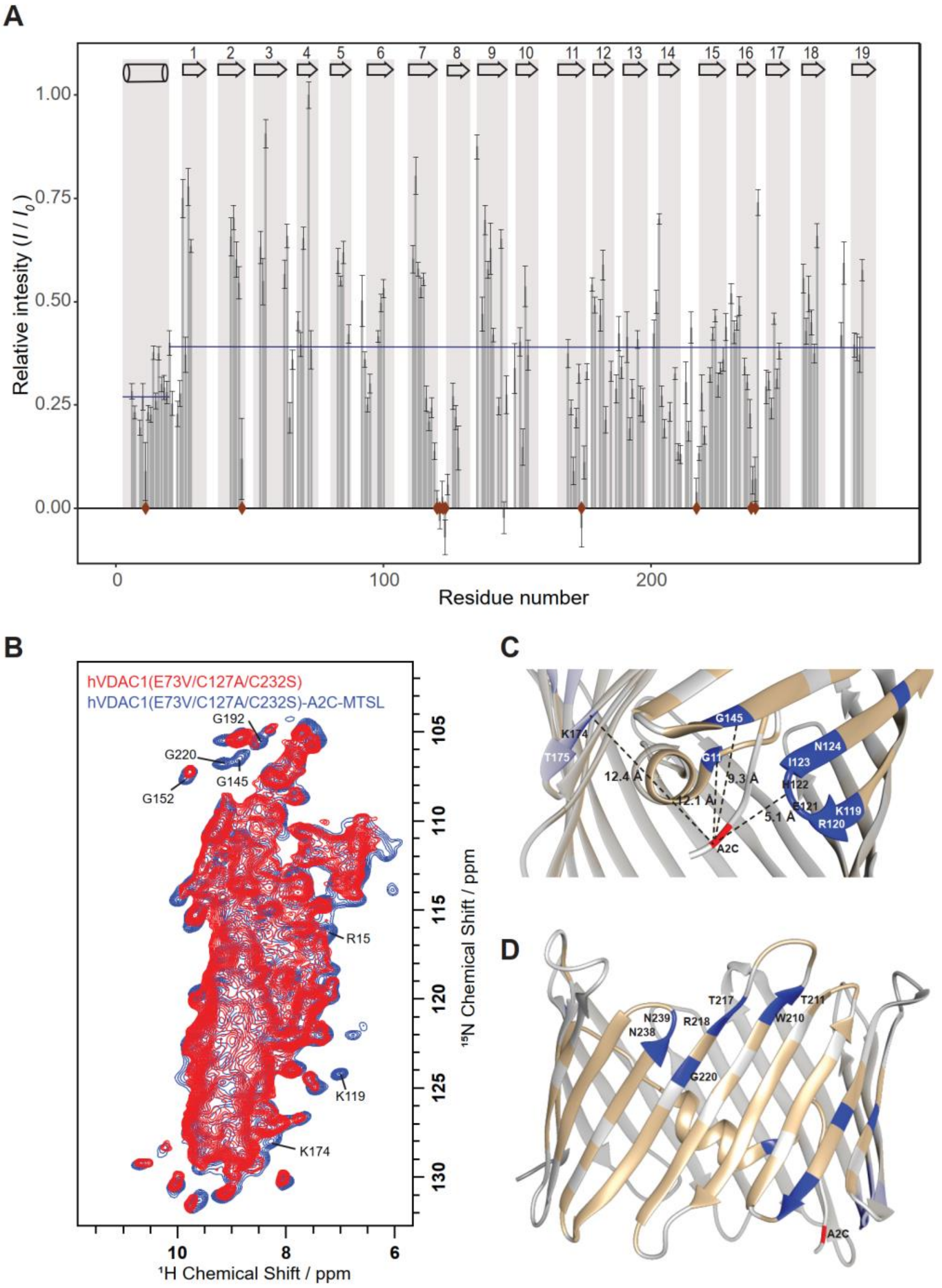

Figure S10: Locating VDAC's N-terminal α-helix with MTSL labeling. A) Intensity changes of hVDAC1(E73VC127AC232S) upon MTSL labeling at the A2C position. Relative intensities were scaled to 1. Red diamonds show residues where intensities were below twice the noise level (no peak was observable). In these cases, the intensities at the expected peak positions are displayed. Average relative intensities in the helix and the barrel are shown by blue lines. B) (H)NH correlation spectrum of hVDAC1 with (red) and without the MTSL label (blue). Resolved peaks broadened beyond detection upon MTSL labeling are assigned. C) and D) Peaks with relative intensities less than $\overline{I_{rel}} - \sigma_{I_{rel}}$ (0.178) are mapped in blue onto PDB 5JDP. The A2C mutation labeled with MTSL is colored in red. Residues that were not assigned or were too overlapped to read off accurate intensity values are colored light grey. C) Landing site of the helix in micelles.[8] Distances to each neighboring loops or β-strand are shown. D) Bleached residues in strands β14-16.

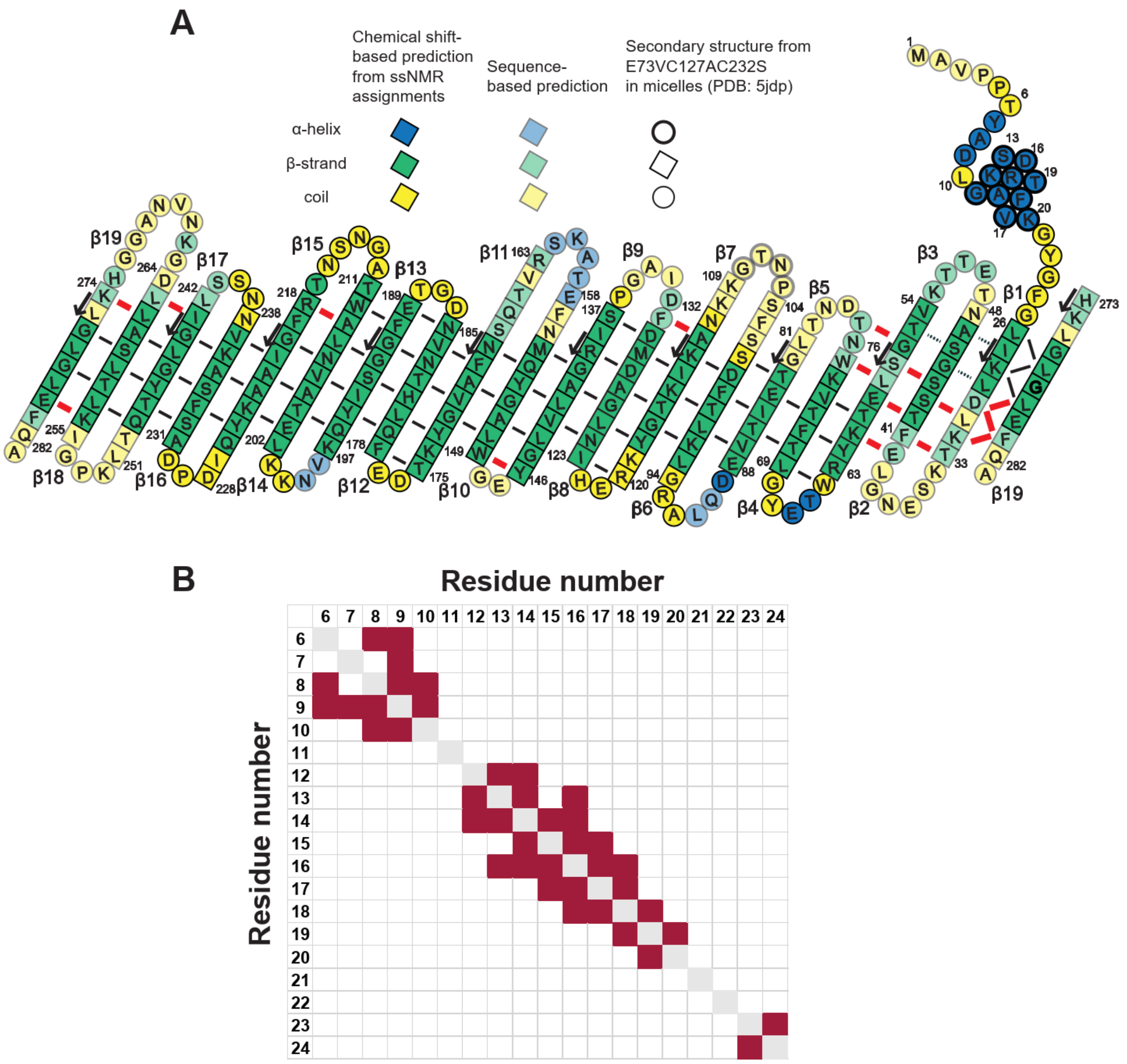

Figure S11: Topology of VDAC in a lipid bilayer as determined by ssNMR A) Long-range, inter β-strand contacts as measured in the HN(H)(H)NH experiment. The hydrogen bonds shown are based on the measured contacts, and are indicated by black lines, ambiguous contacts are shown as dashed lines. Contacts modeled in are shown as red lines. Residues assigned by ssNMR are colored in green. Contacts observed within the barrel are mapped onto the topology model of VDAC(E73V) determined in micelles (PDB: 5jdp). B) Contacts in the N-terminal α-helix, as measured in the HN(H)(H)NH experiment, shown in burgundy. Contacts were mapped symmetrically (i.e. if residue *i* is contacting residue *i+2*, it is indicated on both sides of the diagonal).

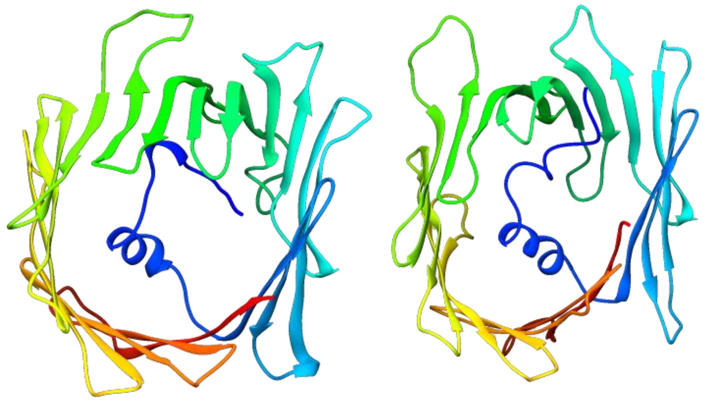

Figure S12: Structures excluded from the ensemble shown on Figure 3. The orientation of strands β5-β10 is not compatible with a planar lipid bilayer.

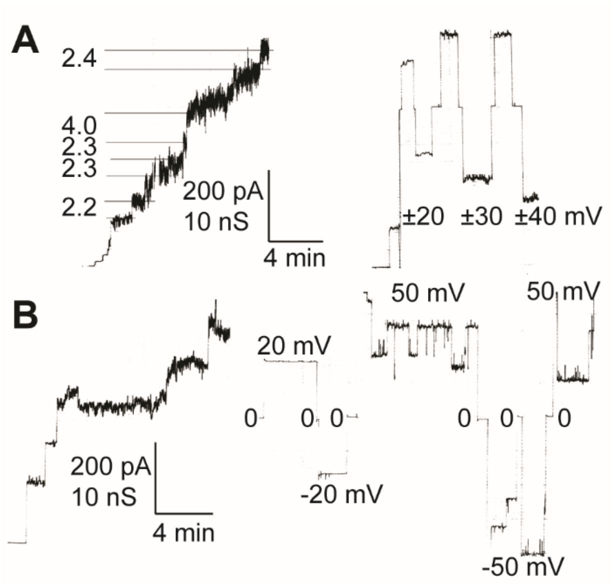

Figure S13: Black lipid membrane characterization of the closed state hVDAC1(G21V/G23V/E73V/C127A/C232S) mutant. (A) Channels with closed-state conductance, not showing gating at higher potentials. (B) Channels showing open state conductance.

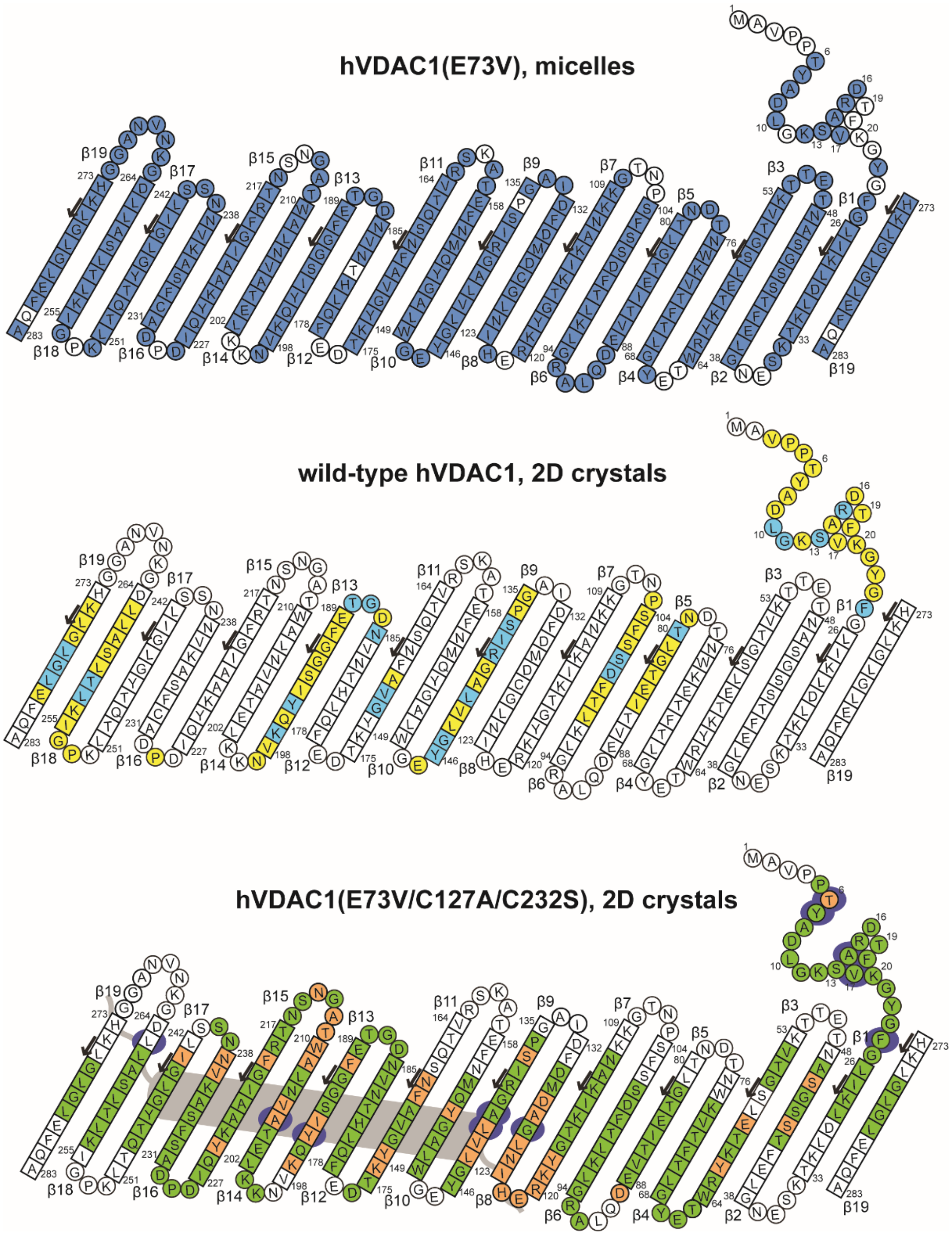

Figure S14: Extent of hVDAC1 assignments. Top, blue: hVDAC1(E73V) assignments in LDAO micelles, solution NMR[11]. Middle, yellow: assignments of wild-type hVDAC1 in 2D crystals, DMPC, ssNMR.[13] Residues colored in light blue show significant chemical shift perturbations compared to our assignments of hVDAC1(E73V/C127A/C232S) in DMPC 2D crystals. Bottom, green: hVDAC1(E73V/C127A/C232S) assignments, in 2D crystals, DMPC, ssNMR. Peach color indicates chemical shift perturbations larger than 0.2 ppm (calculated from H, ^{H}N, Cα, and Cβ shifts, weighted by 1, 0.17, 0.3, and 0.3, respectively).[12, 14] These were calculated from our assignments in 2D crystals, and assignments in LDAO micelles.[15] Squares depict β-sheet, circles helical and loop residues at the N-terminus, and between strands, respectively. Colored fill indicates assigned residue. Blue circles indicate helix-barrel ^{H}N-^{H}N contact sites, as determined by solution NMR.[15] The location of the helix, as determined in solution, is shown in gray.

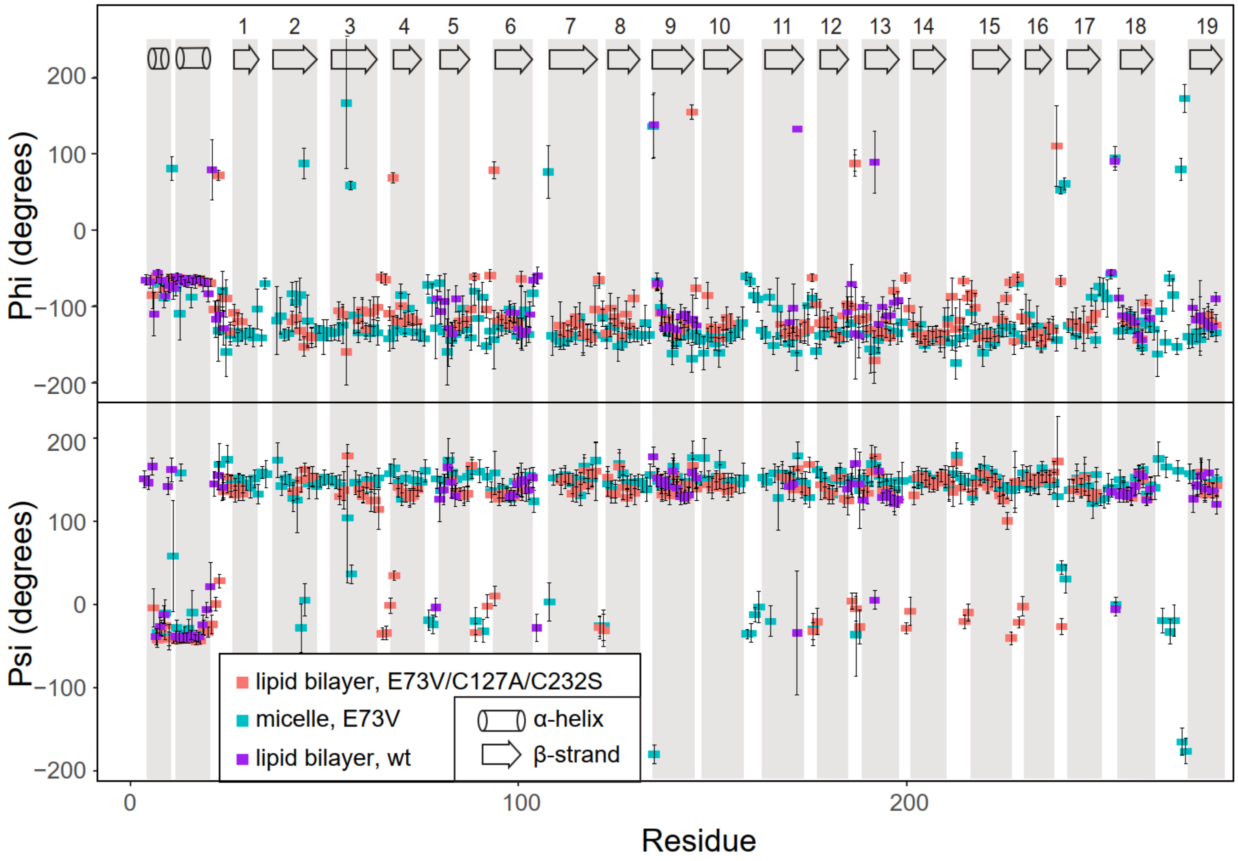

Figure S15: Plot of predicted torsion angles for hVDAC1(E73V/C127A/C232S) in micelles and in a lipid bilayer. Torsion angle predictions were carried out with TALOS-N.[16] Angles based on assignments in a DMPC lipid bilayer, 2D crystals (present study) are shown in blue, while red depicts angle predictions from assignments in LDAO micelles.[8] Purple shows torsion angle predictions of wild-type (wt) hVDAC1 in DMPC 2D crystals, based on assignments by Eddy et al.[13] Secondary structural elements are shown on top and in the figure legend, and are depicted based on the solution NMR structure of the E73V mutant.[8]

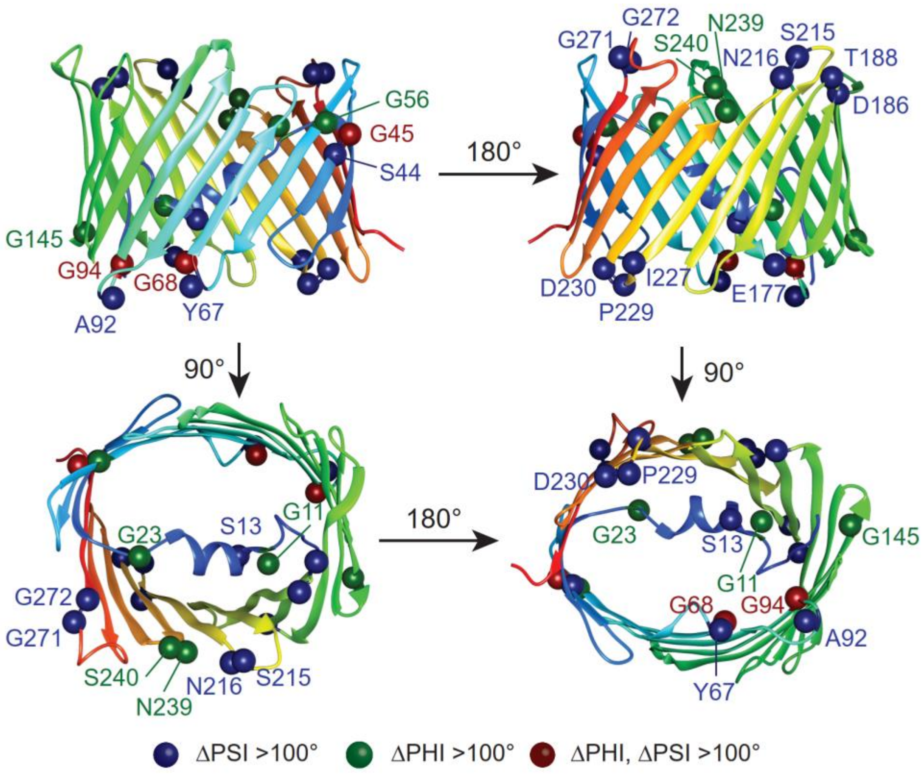

Figure S16: Large discrepancies between predicted torsion angles in micelles and a lipid bilayer for hVDAC1(E73V/C127A/C232S). Differences larger than 100° in phi and psi angles are shown as spheres: green for phi angles, blue for psi angles, red for both phi and psi angles. The protein is colored according to a rainbow scheme, starting with blue at the N-terminus, and finishing with red at the C-terminus. The figure was created using the lowest energy structure of the protein's structure in micelles (PDB: 5jdp)[8] with the Chimera software.[17]

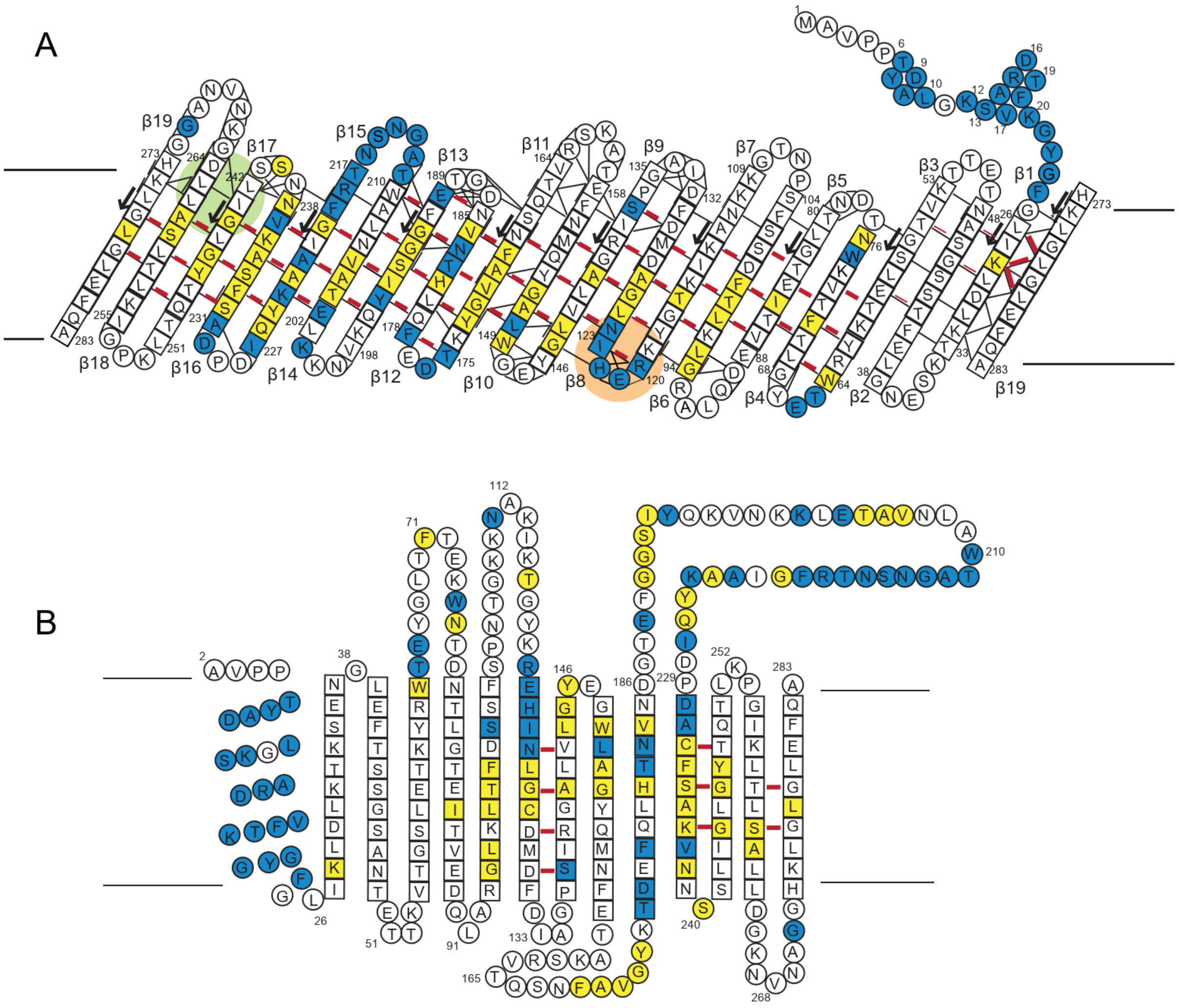

Figure S17: Possible topologies of hVDAC1 in a lipid bilayer. A) Topology model of hVDAC1(E73V) in LDAO micelles from Villinger's thesis.[18] Contacts observed by solution NMR are shown in black lines. (An updated contact map showing more NOE contacts is available,[15] however Villinger's map was chosen for visibility reasons). Contacts observed in hVDAC1(E73V/C127A/C232S) in DMPC 2D crystals are shown in red lines. The helix's landing site is shown with an orange patch, with a possible alternate landing site upon shown in green. B) Topology map of the functional structure suggested by Colombini.[19] Contacts observed in hVDAC1(E73V/C127A/C232S) in DMPC 2D crystals agreeing with the topology proposed by Colombini are shown in red lines. Contacts from z-z mixing experiments[20] are shown in yellow for lipid, and blue for water contacts.

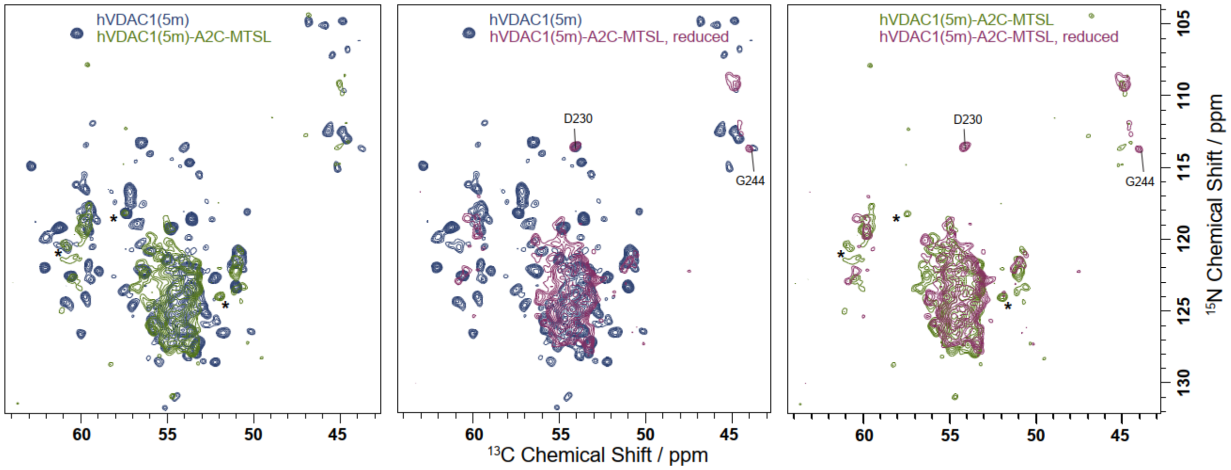

Figure S18: MTSL-labeling of hVDAC1(G21V/G23V/E73V/C127A/C232S) (hVDAC1(5m)-A2C-MTSL)) The ^{13}C-^{15}N projections of (H)CANH spectra are shown for the unlabeled quintuple mutant (blue), the quintuple mutant coupled to an MTSL label at the A2C position (green), and the latter reduced with ascorbic acid (purple). Peaks disappearing upon reduction of the sample are labeled with an asterisk.

CYANA/FLYA library entries for proton-detected MAS experiments. These entries were concatenated to the end of the standard cyana.lib library file for resonance assignment.

SPECTRUM sshCH C H

0.98 C:C_A* H:H_A*

0.2 C:C_A* C_A* H:H_A*

SPECTRUM ssCOcaNH HN N C

0.980 HN:H_AMI N:N_AM* C_ALI C:C_BYL

SPECTRUM ssCOCAnH HN CA C

0.980 HN:H_AMI N_AM* CA:C_ALI C:C_BYL

SPECTRUM ssCOCANH HN N CA C

0.980 HN:H_AMI N:N_AM* CA:C_ALI C:C_BYL

SPECTRUM ssCAcoNH HN N C

0.98 HN:H_AMI N:N_AM* C_BYL C:C_ALI

SPECTRUM ssCACONH HN N C CA

0.98 HN:H_AMI N:N_AM* C:C_BYL CA:C_ALI

SPECTRUM ssCBcaNH HN N C

0.980 HN:H_AMI N:N_AMI (C_BYL) C_ALI C:C_ALI

0.200 HN:H_AMI N:N_AMI C_ALI C:C_ALI

SPECTRUM ssCBcacoNH HN N C

0.980 HN:H_AMI N:N_AMI C_BYL C_ALI (N_AMI) C:C_ALI

0.200 HN:H_AMI N:N_AMI C_BYL C_ALI C:C_ALI

SPECTRUM ssNCAHA HC C N

0.980 HC:H_ALI C:C_ALI N:N_AMI

SPECTRUM ssNcoCAHA HC C N

0.980 HC:H_ALI C:C_ALI C_BYL N:N_AMI

SPECTRUM ssCOnCAHA HC C CO

0.980 HC:H_ALI C:C_ALI N_AMI CO:C_BYL

SPECTRUM ssCBCAHA HC C CB

0.980 HC:H_ALI C:C_ALI CB:C_ALI

SPECTRUM ssCONH HN N C

0.98   HN:H_AMI N:N_AMI C:C_BYL

SPECTRUM ssHhCHbetasheet C H HC

0.980 H:H_AMI N_AMI C_BYL C:C_ALI HC:H_ALI

SPECTRUM ssHhNHbetasheet N H HN

0.980 H:H_ALI C_ALI C_BYL N:N_AMI HN:H_AMI

SPECTRUM sshNcacoNH N2 N1 H1

0.980 N2:N_AMI C_ALI C_BYL N1:N_AMI H1:H_AMI

SPECTRUM sshNcacoCAHA N C HC

0.980 N:N_AMI (C_BYL) C:C_ALI (C_BYL) HC:H_ALI

SPECTRUM sshCANH N C HN

0.980 HN:H_AMI N:N_AMI C:C_ALI C_BYL

SPECTRUM ssCAncoCAHA C2 C1 HC

0.98 H_ALI C2:C_ALI (C_BYL) N_AMI C_BYL C1:C_ALI HC:H_ALI

SPECTRUM ssHNNH H1 N1 N2 H2

0.9 N1:N_AM* H1:H_A* ~4.0 H2:H_A* N2:N_AM*

0.8 N1:N_AM* H1:H_A* ~4.5 H2:H_A* N2:N_AM*

0.7 N1:N_AM* H1:H_A* ~5.0 H2:H_A* N2:N_AM*

0.6 N1:N_AM* H1:H_A* ~5.5 H2:H_A* N2:N_AM*

0.5 N1:N_AM* H1:H_A* ~6.0 H2:H_A* N2:N_AM*

References

(1) Schneider, R.; Etzkorn, M.; Giller, K.; Daebel, V.; Eisfeld, J.; Zweckstetter, M.; Griesinger, C.; Becker, S.; Lange, A., The native conformation of the human VDAC1 N terminus. *Angew. Chem. Int. Ed. Engl.* **2010,** *49*, 1882-1885.

(2) Eddy, M. T.; Andreas, L.; Teijido, O.; Su, Y.; Clark, L.; Noskov, S. Y.; Wagner, G.; Rostovtseva, T. K.; Griffin, R. G., Magic angle spinning nuclear magnetic resonance characterization of voltage-dependent anion channel gating in two-dimensional lipid crystalline bilayers. *Biochemistry* **2015,** *54*, 994-1005.

(3) Goncalves, R. P.; Buzhynskyy, N.; Prima, V.; Sturgis, J. N.; Scheuring, S., Supramolecular assembly of VDAC in native mitochondrial outer membranes. *J. Mol. Biol.* **2007,** *369*, 413-418.
(4) Krause, R. H., ML; Nielsen, JE; Vriend, G WHAT IF: Find most average NMR structure. https://swift.cmbi.umcn.nl/servers/html/bestml.html.

(5) Hiller, S.; Garces, R. G.; Malia, T. J.; Orekhov, V. Y.; Colombini, M.; Wagner, G., Solution structure of the integral human membrane protein VDAC-1 in detergent micelles. *Science* **2008,** *321*, 1206-1210.

(6) Bayrhuber, M.; Meins, T.; Habeck, M.; Becker, S.; Giller, K.; Villinger, S.; Vonrhein, C.; Griesinger, C.; Zweckstetter, M.; Zeth, K., Structure of the human voltage-dependent anion channel. *Proc. Natl. Acad. Sci. U. S. A.* **2008,** *105*, 15370-15375.

(7) Ujwal, R.; Cascio, D.; Colletier, J. P.; Faham, S.; Zhang, J.; Toro, L.; Ping, P.; Abramson, J., The crystal structure of mouse VDAC1 at 2.3 A resolution reveals mechanistic insights into metabolite gating. *Proc. Natl. Acad. Sci. U. S. A.* **2008,** *105*, 17742-17747.

(8) Jaremko, M.; Jaremko, L.; Villinger, S.; Schmidt, C. D.; Griesinger, C.; Becker, S.; Zweckstetter, M., High-Resolution NMR Determination of the Dynamic Structure of Membrane Proteins. *Angew. Chem. Int. Ed. Engl.* **2016,** *55*, 10518-10521.

(9) Hosaka, T.; Okazaki, M.; Kimura-Someya, T.; Ishizuka-Katsura, Y.; Ito, K.; Yokoyama, S.; Dodo, K.; Sodeoka, M.; Shirouzu, M., Crystal structural characterization reveals novel oligomeric interactions of human voltage-dependent anion channel 1. *Protein Sci.* **2017,** *26*, 1749-1758.

(10) Martynowycz, M. W.; Khan, F.; Hattne, J.; Abramson, J.; Gonen, T., MicroED structure of lipid-embedded mammalian mitochondrial voltage-dependent anion channel. *Proc Natl Acad Sci U S A* **2020,** *117*, 32380-32385.

(11) Bohm, R.; Amodeo, G. F.; Murlidaran, S.; Chavali, S.; Wagner, G.; Winterhalter, M.; Brannigan, G.; Hiller, S., The Structural Basis for Low Conductance in the Membrane Protein VDAC upon beta-NADH Binding and Voltage Gating. *Structure* **2020,** *28*, 206-214.

(12) Williamson, M. P., Using chemical shift perturbation to characterise ligand binding. *Progress in nuclear magnetic resonance spectroscopy* **2013,** *73*, 1-16.

(13) Eddy, M. T.; Su, Y.; Silvers, R.; Andreas, L.; Clark, L.; Wagner, G.; Pintacuda, G.; Emsley, L.; Griffin, R. G., Lipid bilayer-bound conformation of an integral membrane beta barrel protein by multidimensional MAS NMR. *J. Biomol. NMR* **2015,** *61*, 299-310.

(14) Long, D.; Yang, D. W., Buffer Interference with Protein Dynamics: A Case Study on Human Liver Fatty Acid Binding Protein. *Biophys. J.* **2009,** *96*, 1482-1488.

(15) Schmidt, C. D. NMR spectroscopic investigations on VDAC. PhD thesis, University of Göttingen, Göttingen, 2017.

(16) Shen, Y.; Bax, A., Protein backbone and sidechain torsion angles predicted from NMR chemical shifts using artificial neural networks. *J. Biomol. NMR* **2013,** *56*, 227-241.

(17) Pettersen, E. F.; Goddard, T. D.; Huang, C. C.; Couch, G. S.; Greenblatt, D. M.; Meng, E. C.; Ferrin, T. E., UCSF Chimera--a visualization system for exploratory research and analysis. *J. Comput. Chem.* **2004,** *25*, 1605-1612.

(18) Villinger, S. Dynamics and interactions of the voltage-dependent anion channel 1 studied by NMR spectroscopy. PhD thesis, University of Göttingen, Göttingen, 2012.

(19) Colombini, M., VDAC: the channel at the interface between mitochondria and the cytosol. *Mol. Cell. Biochem.* **2004,** *256-257*, 107-115.

(20) Najbauer, E. E.; Movellan, K. T.; Schubeis, T.; Schwarzer, T.; Castiglione, K.; Giller, K.; Pintacuda, G.; Becker, S.; Andreas, L. B., Probing Membrane Protein Insertion into Lipid Bilayers by Solid-State NMR. *ChemPhysChem* **2019,** *20*, 302-310.
